# High carrier mobility along the [111] orientation in Cu_2_O photoelectrodes

**DOI:** 10.1038/s41586-024-07273-8

**Published:** 2024-04-24

**Authors:** Linfeng Pan, Linjie Dai, Oliver J. Burton, Lu Chen, Virgil Andrei, Youcheng Zhang, Dan Ren, Jinshui Cheng, Linxiao Wu, Kyle Frohna, Anna Abfalterer, Terry Chien-Jen Yang, Wenzhe Niu, Meng Xia, Stephan Hofmann, Paul J. Dyson, Erwin Reisner, Henning Sirringhaus, Jingshan Luo, Anders Hagfeldt, Michael Grätzel, Samuel D. Stranks

**Affiliations:** 1 Department of Chemical Engineering and Biotechnology, University of Cambridge, Cambridge, UK; 2 Cavendish Laboratory, University of Cambridge, Cambridge, UK; 3 Laboratory of Photonics and Interfaces, Institute of Chemical Sciences and Engineering, École Polytechnique Fédérale de Lausanne, Lausanne, Switzerland; 4 Laboratory of Photomolecular Science, Institute of Chemical Sciences and Engineering, École Polytechnique Fédérale de Lausanne, Lausanne, Switzerland; 5 Department of Engineering, University of Cambridge, Cambridge, UK; 6 Laboratory of Organometallic and Medicinal Chemistry, Institute of Chemical Sciences and Engineering, École Polytechnique Fédérale de Lausanne, Lausanne, Switzerland; 7 Yusuf Hamied Department of Chemistry, University of Cambridge, Cambridge, UK; 8 Institute of Photoelectronic Thin Film Devices and Technology, State Key Laboratory of Photovoltaic Materials and Cells, Ministry of Education Engineering Research Centre of Thin Film Photoelectronic Technology, Renewable Energy Conversion and Storage Centre, Frontiers Science Center for New Organic Matter, Nankai University, Tianjin, China; 9 Department of Chemistry-Ångström Laboratory, Uppsala University, Uppsala, Sweden

**Keywords:** Devices for energy harvesting, Solar fuels

## Abstract

Solar fuels offer a promising approach to provide sustainable fuels by harnessing sunlight^1,2^. Following a decade of advancement, Cu_2_O photocathodes are capable of delivering a performance comparable to that of photoelectrodes with established photovoltaic materials^3–5^. However, considerable bulk charge carrier recombination that is poorly understood still limits further advances in performance^6^. Here we demonstrate performance of Cu_2_O photocathodes beyond the state-of-the-art by exploiting a new conceptual understanding of carrier recombination and transport in single-crystal Cu_2_O thin films. Using ambient liquid-phase epitaxy, we present a new method to grow single-crystal Cu_2_O samples with three crystal orientations. Broadband femtosecond transient reflection spectroscopy measurements were used to quantify anisotropic optoelectronic properties, through which the carrier mobility along the [111] direction was found to be an order of magnitude higher than those along other orientations. Driven by these findings, we developed a polycrystalline Cu_2_O photocathode with an extraordinarily pure (111) orientation and (111) terminating facets using a simple and low-cost method, which delivers 7 mA cm^−2^ current density (more than 70% improvement compared to that of state-of-the-art electrodeposited devices) at 0.5 V versus a reversible hydrogen electrode under air mass 1.5 G illumination, and stable operation over at least 120 h.

## Abstract (web summary)

A study introduces a novel method to grow single-crystal Cu_2_O thin films with selected crystal orientations, highlighting enhanced bulk carrier mobility and carrier diffusion length along the [111] direction that yields Cu_2_O photocathodes with improved performance.

## Main

In the accelerated transition to sustainable energy, solar fuels provide a promising approach to store intermittent solar energy in energy-dense molecules that can usefully be consumed in the existing infrastructure. These processes rely on effective photoinduced charge separation and transport in suitable photoactive materials. In photocatalytic systems, research efforts primarily focus on surface and interface engineering to optimize charge separation efficiencies, owing to built-in electric fields residing at surfaces and typically short charge transporting distances in particles^7,8^. By contrast, state-of-the-art photoelectrochemical (PEC) devices utilize solid-state junctions to efficiently separate electrons and holes, leaving charge transport as a vital issue in achieving high performance^5,9^. In such systems, bulk carrier recombination processes are identified as the key performance bottlenecks, in which a substantial fraction of photoexcited carriers are lost before being injected into the surface catalysts^6^; such processes remain poorly understood but will be essential to push these technologies forwards.

Single-crystal thin films are widely applied in the semiconductor industry^10,11^. The absence of grain boundaries and a substantial decrease in defect density compared with polycrystalline analogues offer distinctive optoelectronic properties. A single-crystal semiconductor is also an ideal platform for detailed spectroscopic studies owing to the ordered and well-defined nature of such structures. Nonetheless, most studies on thin-film semiconductors are carried out with polycrystalline materials owing to the demanding preparation processes for single crystals that often involve high vacuum levels, high temperatures and sophisticated instruments^12^.

Here we demonstrate liquid-phase epitaxial growth of unique single-crystal Cu_2_O (SC-Cu_2_O) thin films under ambient conditions resulting in extremely high-quality crystals with tunable crystal orientations. These unique single-crystal films enable optoelectronic and PEC characterization in steady-state conditions and with a customized femtosecond transient reflection spectroscopy technique spanning the ultraviolet to infrared spectral regions in three crystal orientations. Our collective results show that the mobility, conductivity and carrier diffusion length along the [111] direction are much greater compared with those in other directions in Cu_2_O, which are essential bulk charge transport properties for solar fuels, photovoltaics, transistors and detectors. Building on these fundamental insights, we fabricated polycrystalline Cu_2_O with an extraordinarily pure (111) orientation and (111) terminating facets using a simple and low-cost method, achieving a Cu_2_O PEC performance beyond that of the state-of-the-art.

## SC-Cu_2_O thin films

**Fig. 1:**
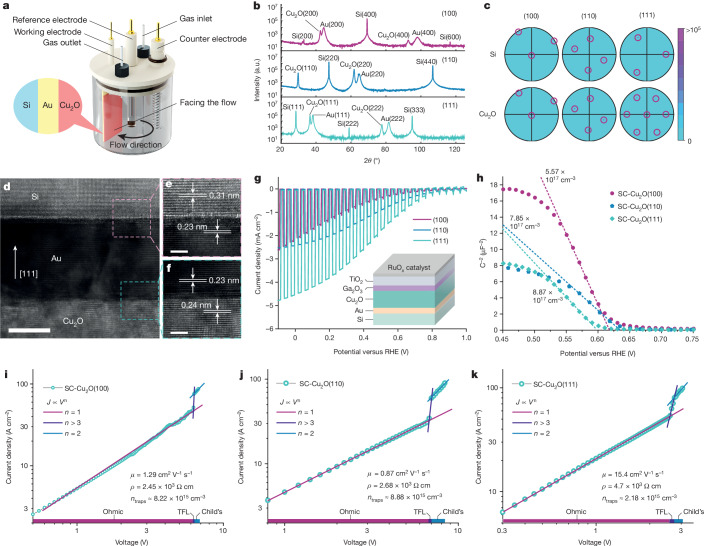
SC-Cu_2_O thin films show anisotropic PEC performance and mobilities. **a**, The electrochemical epitaxy set-up with the three-electrode configuration for liquid-phase epitaxial growth of Cu_2_O thin films. **b**,**c**, X-ray diffraction patterns (**b**) and pole figures generated from EBSD (**c**) for single-crystal epitaxial layers on Si substrates with reflections highlighted in circles. a.u., arbitrary units. **d**–**f**, A cross-sectional transmission electron micrograph showing Cu_2_O(111)/Au(111)/Si(111) layers (**d**), with close-up images at the Cu_2_O/Au (**f**) and Au/Si (**e**) interfaces, with lattice spacings in agreement with bulk references. Scale bars, 10 nm (**d**) and 2 nm (**e**,**f**). **g**, PEC responses of SC-Cu_2_O photocathodes for solar hydrogen evolution under a simulated one-sun condition. The inset shows the layered structure of the Cu_2_O photocathodes. **h**, Mott–Schottky plots of SC-Cu_2_O thin films tested in pH 9 carbonate buffer solution. The carrier densities were calculated based on the slopes of the linear fitting represented by the dotted lines. **i**–**k**, Current density–voltage curves of hole-only devices with SC-Cu_2_O(100) (**i**), SC-Cu_2_O(110) (**j**) and SC-Cu_2_O(111) (**k**).

The Cu_2_O and Au proxy layers were grown by liquid-phase epitaxy at room temperature using the three-electrode configuration shown in Fig. 1a. To achieve high-quality films, the substrates need to be fixed opposite to the flow with controlled velocity (Methods). Thin Au buffer layers were first grown on degenerate Si substrates with three selected orientations, followed by deposition of Cu_2_O (Supplementary Figs. 1 and 2). The out-of-plane orientations of each layer were determined by X-ray diffraction, as shown in Fig. 1b and Supplementary Fig. 3. Even with the intensity being viewed on the logarithmic scale, only the expected out-of-plane orientation is detected for each pattern, indicating the exceptional orientation purity of the samples. The in-plane orientations were confirmed by electron backscatter diffraction (EBSD) pole figures (Fig. 1c, with corresponding inverse pole figures and *z*-direction inverse pole figure maps in Supplementary Figs. 4 and 5). Distinct spots occur at the expected azimuthal angles for each given orientation owing to the Bragg condition being satisfied in films with in-plane order^13^. Samples with (100) and (110) orientations show SC-Cu_2_O features over all areas, whereas the Cu_2_O(111) film is shown to be twinned, with two Cu_2_O(111) crystalline domains rotated 60° to each other. Cross-sectional transmission electron microscopy was implemented on focused ion beam-thinned lamellae to image the interfaces of the Cu_2_O layer electrodeposited onto the Au buffer layer on a Si(111) substrate (Fig. 1d). The Au section is less transparent owing to the higher resistance against focused ion beam etching during the sampling process. The lattice spacing of each layer was calculated by measuring the distance of 27 lattice lines under the grey value condition (Supplementary Figs. 6–8) and is marked in the close-up images in Fig. 1e,f. Spacings of 0.24 and 0.23 nm were measured for Cu_2_O and Au, respectively. The same cubic structure and small lattice mismatch of 4.3% favour successful epitaxy with moderate compressive in-plane strain. The coincident site lattices in which 4 unit meshes of Au coincide with 3 unit meshes of Si allowed the epitaxy at the Au/Si interface even although the nominal lattice mismatch calculated from Fig. 1e is −25.8% (ref. ^13^). Additionally, the epitaxial relation between layers on the SC-Cu_2_O(100) sample was also confirmed by cross-sectional transmission electron microscopy (Supplementary Fig. 9). These results collectively provide direct evidence of epitaxial growth of high-quality, aligned Cu_2_O on Si wafers using Au as the buffer layer.

Intriguing PEC behaviour was discovered by testing the photocathodes prepared using SC-Cu_2_O thin films of different orientations (Fig. 1g) in the illustrated architecture (inset in Fig. 1g). Although all photoelectrodes exhibit similar photovoltages, determined from the same buried p–n junction^3^, the SC-Cu_2_O(111) device delivers a much higher current density of 4.5 mA cm^−2^ at 0 V versus the reversible hydrogen electrode (RHE), a factor of two more than those of the other orientations. The scalability of the epitaxial method was demonstrated by fabricating a wafer-size SC-Cu_2_O(110) photocathode with an active area of 60 cm^2^ (Supplementary Fig. 10). To understand the anisotropic PEC performance, the orientation-dependent out-of-plane carrier transport properties were investigated. Electrochemical impedance spectroscopy at the semiconductor/electrolyte interface was used to determine the carrier densities and flat band potentials by fitting the Mott–Schottky function (Fig. 1h for 1 kHz; similar results were seen for other frequencies (Supplementary Fig. 11 and Supplementary Table 3)). Although all films demonstrate p-type character and carrier densities of the order of 10^17^ cm^−3^, which is similar to that of thermally prepared Cu_2_O thin films^14,15^, no apparent carrier density anisotropy was observed. The same finding also applies to the orientation-dependent flat band potentials, which are 0.62, 0.60 and 0.63 V versus the RHE for SC-Cu_2_O(100), SC-Cu_2_O(111) and SC-Cu_2_O(110), respectively.

To investigate the carrier mobility, hole-only devices were built with SC-Cu_2_O sandwiched between Au and MoO_*x*_/Ag layers (Methods) and space-charge-limited current analyses were carried out^16,17^. Figure 1i–k shows the *j*–*V* relation of SC-Cu_2_O films on a double logarithmic scale. The clear quadratic dependence of the current on the applied voltage is observed in the Child’s law region. From this region, we extract carrier mobilities of 1.29, 0.87 and 15.4 cm^2^ V^−1^ s^−1^ for SC-Cu_2_O(100), SC-Cu_2_O(110) and SC-Cu_2_O(111), respectively, with the Mott–Gurney law (Methods, Supplementary Figs. 12–14 and Supplementary Tables 1 and 2). The anisotropic hole mobilities are reasonable, as the effective hole masses are found to be highly orientation dependent for cubic Cu_2_O (refs. ^18,19^). The anisotropy in mobility correlates well with the anisotropic PEC performance, with the highest mobility along [111] being more than one order of magnitude higher than those of the other orientations, and the PEC current being a factor of two larger. Remarkably, the SC-Cu_2_O hole mobility measured here for samples fabricated in ambient conditions is comparable to those of Cu_2_O thin films prepared using methods involving high temperatures, vacuum processing or doping^15,20,21^.

The orientation-dependent resistivities were also calculated from the linear ohmic region. The rapid rise of current with increased voltage in the (111)-oriented samples (Fig. 1k) yields the lowest resistivity of 4.7 × 10^2^ Ω cm, which makes [111] the most conductive direction among all. We rule out anisotropic contact resistivities between Au and Cu_2_O as a dominant influence on device performance as the contact resistivities of 39.2, 7.25 and 17.4 mΩ cm^2^ for SC-Cu_2_O(100), SC-Cu_2_O(110) and SC-Cu_2_O(111), respectively, determined by a modified transfer length method (Supplementary Fig. 15), did not follow device trends. These results are in good agreement with the findings of an anisotropic resistivity study on Cu_2_O nanoparticles^22^. The in-gap trap states were also studied to help unveil the physical origin of the performance and electronic properties. The trap-filled limit voltage (*V*_TFL_) is located at the transition where the average density of injected holes becomes comparable to the material hole density in thermal equilibrium, and excess injected holes push the quasi-Fermi level towards the valence band, completely ionizing the donor-type hole traps (see comparison in Supplementary Fig. 16). We found the lowest trap density of 2.18 × 10^15^ cm^−3^ for the SC-Cu_2_O(111) (8.88 × 10^15^ cm^−3^ for SC-Cu_2_O(110) and 8.22 × 10^15^ cm^−3^ for SC-Cu_2_O(100)), which points to a less defective electronic structure comparable to those of a wide range of established and emerging photovoltaic-grade materials such as polycrystalline Si and hybrid perovskites^10–12^. Combining the results above (summarized in Supplementary Table 4; photoluminescence in Supplementary Fig. 17), we find that anisotropic carrier mobility and trap behaviour play a vital role in orientation-dependent PEC performance.

## Ultrafast carrier dynamics

**Fig. 2:**
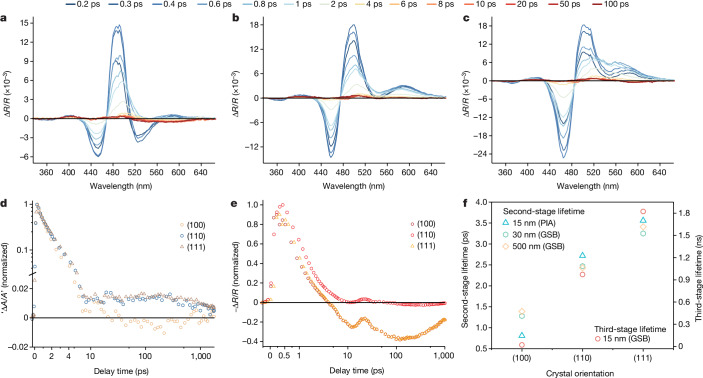
Transient reflection spectroscopy on SC-Cu_2_O films with various crystal orientations. **a**–**c**, Transient reflection spectra of 15-nm Cu_2_O films with crystal orientations of (100) (**a**), (110) (**b**) and (111) (**c**). Pump wavelength, 400 nm; fluence, 110 µJ cm^−2^. **d**,**e**, Kinetics of the orientation-dependent photoinduced ground state bleach signals from the reproduced transient absorption spectra (450–490 nm for the (100) orientation, 460–500 nm for the (110) orientation, and 470–510 nm for the (111) orientation; Supplementary Fig. 19) (**d**), and the photoinduced absorption (600–650 nm; Supplementary Fig. 18) from the transient reflection spectra (**e**) of SC-Cu_2_O. **f**, Second-stage and third-stage lifetime of SC-Cu_2_O films with various thicknesses. PIA, photoinduced absorption; GSB, ground-state bleach.

To further understand the relationship between orientation and performance, the carrier dynamics of SC-Cu_2_O films were examined using ultrafast transient reflection spectroscopy across a wide spectral range. Figure 2a–c and Supplementary Fig. 18 show transient reflection spectra and maps in the ultraviolet–visible region for 15-nm-thick Cu_2_O thin films of three orientations under a 3.1-eV excitation pump with a fluence of about 110 µJ cm^−2^, corresponding to a carrier density of about 6.5 × 10^19^ cm^3^ (see set-up illustration in Supplementary Fig. 18m). An antisymmetric spectral pattern comprising two peaks at about 2.70 and about 2.48 eV was observed for all samples, with an isosbestic point at about 2.58 eV where the transient reflection intensity does not change with pump–probe delay. Another noticeable feature in the response is the shoulder peak at the lower-energy side of the antisymmetric peak. The transient absorption spectrum of the system can be derived from the inverse Hilbert transform of the experimentally measured transient reflection spectrum^23^, with the pair of antisymmetric peaks in the transient reflection spectra corresponding to a negative symmetric peak centred at about 2.58 eV in the calculated transient absorption spectra. The features at the lower-energy side correspond to a weak broadband response that exhibits a much more pronounced positive component (Supplementary Fig. 19). The negative signals of the derived transient absorption features represent the bleaching of the transition from the ground state to the excited state (that is, the ground-state bleach), and the positive signals correspond to the photoinduced absorption^24,25^. As a result, we attribute the antisymmetric peaks in the transient reflection spectra to the bleach of the transition and assign the low-energy features to the excited-state absorption (ESA) from the first excited state to higher excited states. To accurately collect the weak ESA signal, the transient reflection was recorded using a visible continuum probe with a pre-split reference beam (Methods) under the same excitation conditions (Supplementary Fig. 18). An ESA signal ranging from 530 to 760 nm was observed for all orientations^24^.

Figure 2d shows the kinetics of the ground-state bleach of Cu_2_O samples with different crystal orientations extracted from the negative peak in the reproduced transient absorption signal (Supplementary Fig. 19). The signal builds up in the pump duration (about 100 fs) and decays with three discernible stages. The first stage is an ultrafast decay on a sub-picosecond timescale, which we ascribe to carrier cooling processes due to carrier–phonon interactions^26^. This stage is followed by a second-stage short-lived component with a lifetime of 1.27, 1.27 and 1.45 ps for the (100), (110) and (111) samples, respectively. This short-lived decay has been assigned to the deactivation of excitons^24,25^. The (111) sample shows the most extended exciton lifetime, consistent with the kinetics of the ESA signal (Fig. 2e), for which the exciton decay lifetime decreases from about 3.6 ps for the (111) sample, to about 2.7 ps for the (110) sample, to about 0.8 ps for the (100) sample extracted from bi-exponential fitting of the ESA signals. The prolonged exciton lifetime for the (111) orientation is further confirmed in Cu_2_O samples of increasing thicknesses (Fig. 2f; see Supplementary Figs. 20 and 21 for details), indicating a lower trap density in the (111) sample as suggested in the steady-state results^25^. Finally, a long-lived component is observed with a lifetime of 20.3, 971.3 and 1,823.4 ps for the (100), (110) and (111) samples, respectively, which is related to carrier recombination processes^24–27^ (the third-stage decay for the 15-nm (110) and (111) samples is enlarged in Supplementary Fig. 22 and lifetimes are marked in Fig. 2f). The longest recombination lifetime in the [111] orientation, combined with the highest hole conductivity, leads to the longest hole diffusion length of *L*_h_^(111)^ = 269.7 nm in the SC-Cu_2_O(111) films, much longer than *L*_h_^(110)^ = 46.8 nm and *L*_h_^(100)^ = 8.2 nm for the (110) and (100) crystal orientations, respectively. Given an effective polaron mass of 0.69*m*_0_ and 0.99*m*_0_ (*m*_0_ is the mass of the free electron) for heavy holes and electrons, respectively^28^, and assuming a similar scattering rate for the holes and electrons, the electron diffusion lengths were calculated as *L*_e_^(111)^ = 188.0 nm, *L*_e_^(110)^ = 32.6 nm and *L*_e_^(100)^ = 5.7 nm. For optoelectronic devices in which thick crystals (>500 nm) are required for optimal light harvesting, a short diffusion length leads to limited currents owing to inefficient carrier extraction. The substantially longer carrier diffusion length in SC-Cu_2_O(111) thus explains the increased current for that orientation and the resulting anisotropic PEC behaviour. Therefore, we expect Cu_2_O(111) thin films would yield the best Cu_2_O optoelectronic devices, benefiting from excellent carrier mobility and long carrier diffusion lengths.

## Optimizing polycrystalline Cu_2_O photoelectrodes

**Fig. 3:**
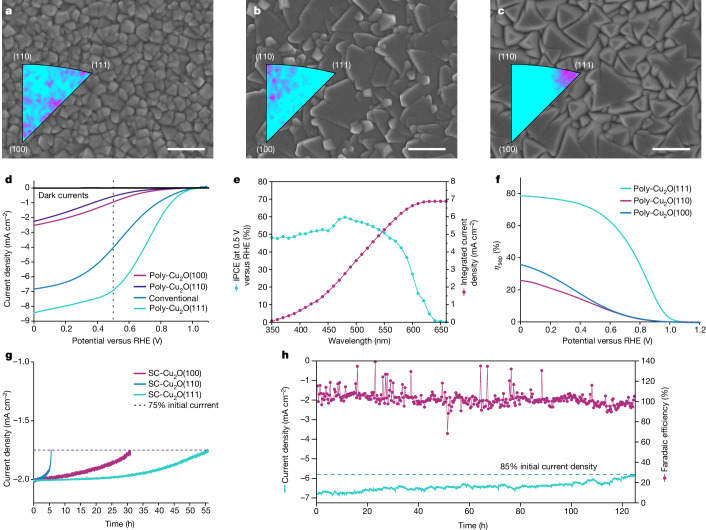
Polycrystalline Cu_2_O photocathodes with preferred orientations. **a**–**c**, Top-view scanning electron micrographs of Cu_2_O polycrystalline thin films with the preferential (100) (**a**), (110) (**b**) and (111) (**c**) orientations and their corresponding inverse pole figures from EBSD. Scale bars, 1 μm. **d**, Current density–potential (*J*–*E*) responses of conventional and polycrystalline Cu_2_O photocathodes with preferred orientations under simulated one-sun AM1.5 G illumination. **e**, Wavelength-dependent IPCE of the poly-Cu_2_O(111) photocathode tested with an applied bias of 0.5 V versus the RHE. **f**, Carrier separation efficiency as a function of applied potential for poly-Cu_2_O photocathodes with three crystal orientations. **g**, Stability test on SC-Cu_2_O photocathodes with a 20-nm TiO_2_ layer biased at various potentials to each deliver an initial current density of 2 mA cm^−2^ (SC-Cu_2_O(100) at 0.03 V versus the RHE, SC-Cu_2_O(110) at 0.21 V versus the RHE, SC-Cu_2_O(111) at 0.48 V versus the RHE) using chronoamperometry techniques. All measurements with SC-Cu_2_O were ceased when the photoelectrodes lost 25% of their initial photocurrent. **h**, Stability test on poly-Cu_2_O(111) with a 100-nm TiO_2_ protection layer at the fixed bias of 0.5 V versus the RHE in pH 5 buffered electrolyte under simulated one-sun illumination (AM1.5 G). After 120 h of operation with H_2_ gas quantification, the initial current density decreased by less than 15%.

To exploit these findings, we fabricated polycrystalline Cu_2_O analogues (poly-Cu_2_O) with preferential (100), (110) and (111) orientations, with the polycrystalline films allowing greater light in-coupling than the more reflective single-crystal samples to demonstrate maximum device performance (Supplementary Discussion 2 and Supplementary Figs. 23–28). Unlike previous performance-enhancing strategies that involve integrating extra layers or high-temperature processing^4,5^, our method was developed to yield specific orientations by simply fine-tuning the pH of the electrolyte; a materials cost estimation for the pilot production of a 1-m^2^ Cu_2_O layer (Supplementary Discussion 3 and Supplementary Tables 5–7), together with the scaled deposition (Supplementary Fig. 10), shows that the electrochemical methods used in this work (that is, liquid-phase epitaxy and electrochemical deposition) are both low cost and scalable. The crystallographic information was acquired by X-ray diffraction analysis, which confirms that the samples demonstrate strong orientation preference (Supplementary Fig. 29). Figure 3a–c shows top-view scanning electron micrographs distinguishing the morphology of well-defined crystals in films of poly-Cu_2_O(100) (three-sided and four-sided pyramids), poly-Cu_2_O(110) (sloping planes) and poly-Cu_2_O(111) (triangle planes). To visualize the degree of (100), (110) and (111) orientation of the Cu_2_O in the polycrystalline films, inverse pole figures were constructed, on the basis of EBSD data (Supplementary Fig. 30), by plotting the orientations with respect to the sample normal (insets of Fig. 3a–c). The preferential distributions towards the (100), (110) and (111) corners show a substantial tendency for the grains to be aligned with the respective orientations, highlighting the particularly extreme orientation purity for the poly-Cu_2_O(111) sample. The EBSD maps corroborate this observation (Supplementary Fig. 31).

The different poly-Cu_2_O films, including the conventional electrodeposited polycrystalline film, were then used to prepare photoelectrodes that were tested under air mass 1.5 G (AM1.5 G), 100 mW cm^−2^ simulated illumination (Methods). We find that poly-Cu_2_O(111) delivers 7 mA cm^−2^ current density at 0.5 V versus the RHE, the value at which Cu_2_O photocathodes operate in most standalone solar water-splitting devices^3,5,29^. This yields a photocurrent density that is a factor of seven higher than that in poly-Cu_2_O(100) (Fig. 3d). All devices in the polycrystalline form achieved a higher current density than their single-crystal analogues owing to the rougher surfaces of polycrystalline films allowing better light in-coupling. The pH-dependent PEC performance was recorded in acidic, neutral and alkaline electrolytes showing slight variation (Supplementary Fig. 32). The wavelength-dependent incident-photon-to-current efficiencies (IPCEs) of poly-Cu_2_O(111) stand out as the highest among all crystal orientations, with an average efficiency exceeding 50% in the bandgap at 0.5 V versus the RHE (Fig. 3e and Supplementary Fig. 33). The integrated product of the IPCE and the photon flux of AM1.5 G yields current densities of 1.46, 0.66 and 6.9 mA cm^−2^, which correlate well with the PEC values. Furthermore, the quantification of charge separation efficiencies, *η*_sep_, is detailed in Supplementary Discussion 4 (also see Methods and Supplementary Figs. 34 and 35). Using Eu^3+^ as an effective electron acceptor, the onset potential of poly-Cu_2_O(111) indicates a built-in photovoltage of beyond 1 V (Fig. 3f). Most importantly, the exceedingly high *η*_sep_ observed in poly-Cu_2_O(111) highlights a substantial advantage of electronic properties along the [111] orientation, excluding other factors concerning light in-coupling and electrocatalysis.

Finally, we investigated how the orientation dependence translates to device operational stability. Comparative stability tests were carried out first on the single-crystal (SC-Cu_2_O) photocathodes with all three orientations by biasing and maintaining the photocathodes at certain potentials to deliver 2 mA cm^−2^ until 25% of the initial current density was lost. SC-Cu_2_O(111) maintained the longest stable operation (>55 h) whereas the SC-Cu_2_O(110) device degraded most quickly (7 h; Fig. 3g), thus establishing that the (111) devices are also the most stable. To assess the longer-term stability with the highest-performing polycrystalline devices, we prepared poly-Cu_2_O photocathodes with 100-nm TiO_2_ overlayers tested in pH 5 buffered electrolyte under simulated one-sun illumination and continuous stirring. The poly-Cu_2_O(111) photocathode delivered extremely stable operation, losing only 15% of its initial photocurrent after 120 h (Fig. 3h). The faradaic efficiency for the hydrogen evolution reaction remained at 100% with a slight decrease at the end, indicating that electrolyte penetration could have occurred (see Supplementary Discussion 5). Indeed, the extended stability test on the examined poly-Cu_2_O(111) revealed an accelerated current decay in the first 20 h followed by protection layer detachment and degradation of current (Supplementary Fig. 36a). Nonetheless, compared to the other two poly-Cu_2_O photocathodes (Supplementary Fig. 36b,c), poly-Cu_2_O(111) demonstrates notably superior stability. Although we acknowledge the crucial role of the TiO_2_ protection layer in stabilizing the photocathodes (Supplementary Fig. 37), it is apparent that the facets exert a substantial impact on the anisotropic stability. The crystal orientations and terminating facets do not inherently have to align, but this particular situation holds true for the poly-Cu_2_O(111) photocathode, for which a high ratio of (111) facets are exposed as seen as in-plane triangles in a cubic system^30^. Stability tests were carried out on fine-tuned bare Cu_2_O thin films, simultaneously showing the (100) and (111) facets (Supplementary Fig. 38a). Under identical testing conditions, the (100) facets experienced considerably greater damage, whereas the (111) facets maintained their flat profile (Supplementary Fig. 38b,c). The exceptional PEC stability aligns with (111) facets being reported to exhibit the lowest surface energy^31,32^. Nevertheless, a comprehensive and meticulous investigation is desirable to consider other potential factors with thorough materials and spectroscopic characterization. In summary, our poly-Cu_2_O(111) photocathodes concurrently demonstrate remarkable PEC performance and stability due to inhibited bulk recombination along the [111] direction and extensive exposure of (111) facets. The performance of 7 mA cm^−2^ current density at 0.5 V versus the RHE represents a 75% improvement compared with the state-of-the-art electrodeposited devices and 15% compared with nanowire systems even although nanowires have intrinsic light management benefits. These important technological advances validate our findings of the anisotropic optoelectronic and PEC properties in Cu_2_O.

## Discussion

The powerful combination of unique SC-Cu_2_O thin films with selected orientations, customized ultrafast spectroscopy and device characterization has resulted in a step change in understanding anisotropic bulk carrier transport properties. To exploit the findings of excellent carrier mobility and carrier diffusion length along [111] in Cu_2_O, poly-Cu_2_O photocathodes with an extraordinarily pure (111) orientation (optimizing bulk recombination, which otherwise limits performance) and (111) terminating facets (optimizing surface effects, which otherwise limit stability) were realized by a simple and low-cost method, delivering a PEC current density of 7 mA cm^−2^ at 0.5 V versus the RHE and more than 120 h of stable operation.

These results enable a new level of precision in future design of high-performance Cu_2_O devices. For example, nanowire Cu_2_O photoelectrodes with an axis parallel to the (111) crystal orientation and optimal radius, considering efficient light absorption and charge collection based on specific carrier diffusion lengths, should be targeted. Further optimizing p–n junctions, applying textured substrates, passivation approaches and photonics, while retaining both bulk and surface directionality properties, will be critical steps for further improvements of performance and stability of these device structures. The demonstration of understanding and exploitation of the anisotropy in thin films presented here provides a widely applicable strategy to cope with the poor bulk carrier transport nature of oxides in their applications in photovoltaics, transistors and detectors, in addition to solar fuels.

## Methods

### Preparation of the Cu_2_O thin films

Si wafers of (100), (110) and (111) orientations without photoelectric function were used as the substrates for epitaxy. All wafers are phosphorus-doped with a resistivity of less than 0.001 Ω cm (Sil’tronix ST). To remove organic contamination on the surface, the wafers were sonicated in warm acetone, cold methanol and deionized water baths in sequence for 10 min each and blow-dried with nitrogen between each bath. Then 5% hydrofluoric acid solution (extreme caution and professional training should be exercised with this highly corrosive and volatile acid) was used to dissolve the native oxide layer and create a hydrogen-terminated surface. After 5 min dipping, the wafer was thoroughly rinsed with deionized water and installed on the electrodeposition set-up directly. The gold buffer layer was electrodeposited in a modified epitaxial method previously designed for epitaxial lift-off procedures^13^. Before immersing the substrates into electrolyte containing 0.06 mM HAuCl_4_, 1 mM KCl, 1 mM H_2_SO_4_ (for stabilizing the Au precursor) and 20 mM K_2_SO_4_ (for enhancing the electrolyte conductivity), the cathode was biased at −1.9 V versus a Ag/AgCl reference electrode. The electrolyte was saturated with N_2_ by 1 h of bubbling and continuous bubbling during film growth. To guarantee the uniformity of the gold layer, the cathode was adjusted to face the electrolyte current with approximately 1.5 cm to the Pt coil counter electrode and 2 mm to the reference electrode. The growth was carried out in a 250-ml container, with stirring at 150 r.p.m. In a typical gold deposition, the polarization is maintained for 20 min. The Cu_2_O epitaxy was developed from previous methods^4,33^, and subsequently carried out in a buffered copper sulphate solution while using the Au epitaxial configuration. A 7.98 g quantity of Cu_2_SO_4_, 21.77 g K_2_HPO_4_ and 67.5 g lactic acid were dissolved in 250 ml H_2_O and then the pH of the solution was adjusted to 12 by adding 2 M KOH solution, thereby yielding a final solution volume of 1 l. The Cu_2_O layer was deposited by chronopotentiometry in a cylinder cell with an 8 cm diameter with continuous stirring (200 r.p.m.). The electrolyte was bubbled with N_2_ throughout the growth. The cathodic deposition was carried out in a two-electrode configuration with a Pt counter electrode using a current density of −0.02 mA cm^−2^. The parallel working electrode and counter electrode are separated by a distance of approximately 0.8 cm, and their respective areas have an approximate ratio of 1:5. The deposition duration varies, and all samples were rinsed with copious deionized water before drying. Polycrystalline Cu_2_O with preferential orientations was electrodeposited in the above-mentioned buffered copper sulphate solution on Au/FTO substrates. A 300-nm layer of polycrystalline Au was sputtered onto FTO substrates cleaned by acetone, ethanol and detergent under sonication. Poly-Cu_2_O(100), poly-Cu_2_O(110) and poly-Cu_2_O(111) were prepared in pH 9.0, 9.6 and 12.6 solutions, respectively. All pH values in the specific electrolytes were measured and tuned three times at 1-h intervals, resulting in a volume of 1 l. Electrodeposition was carried out with these solutions after overnight stirring using a constant −0.1 mA cm^−2^ current density. Thin films for photocathodes had an electrodeposition duration of 100 min followed by a thorough rinse with copious deionized water and drying with an air gun.

### Fabrication of the photocathodes

To examine the PEC performance of the photoelectrodes with various types of Cu_2_O, atomic layer deposition was applied to build the buried p–n junction^3^. Directly after the epitaxial layers, Ga_2_O_3_ was deposited at a substrate temperature of 150 °C using bis(μ-dimethylamino)tetrakis(dimethylamino)digallium (98%; Strem Chemicals) as the precursor and deionized water as the oxidant. The Ga precursor was preheated to 125 °C to maintain a sufficient vapour pressure. In every deposition cycle, 0.5 s precursor pulsing time, 0.05 s water pulse and 15 s pumping time were programmed with a constant nitrogen carrier gas of 10 sccm. For all Cu_2_O photocathodes, the deposition cycle number is set to 135, resulting in a Ga_2_O_3_ layer of 20 nm. A TiO_2_ layer was then coated to protect the photocathode against electrolyte corrosion using tetrakis(dimethylamino)titanium (99.999%; Sigma) as the metal precursor and deionized water as the oxidant. The chamber temperature remained at 150 °C whereas the precursor was heated to 75 °C. Each cycle consisted of 0.1 s precursor pulsing time, 0.05 s water pulse and 15 s pumping time. A total of 340 cycles of TiO_2_ deposition were applied, resulting in a TiO_2_ layer of 20 nm. The polycrystalline devices for stability tests use 100 nm TiO_2_ as a protection layer. A RuO_*x*_ hydrogen evolution catalyst was used to extract photo-generated electrons for water reduction. Briefly, the deposition was carried out in a 1.3 mM KRuO_4_ solution at a current density of −10 μA cm^−2^ under simulated one-sun illumination. Each catalyst decoration required 6 min, and a platinum wire was used as the counter electrode.

### Materials characterization

The crystal information of the Cu_2_O films was characterized by X-ray diffraction on an Empyrean system (PANalytical) with a PIXcel-1D detector and Cu Kα radiation. Diffraction patterns were recorded at a scan rate of 1° min^−1^ with a step width of 0.02° using a two-bounce hybrid monochromator and parallel-plate collimator diffracted optics. High-resolution scanning electron microscopy was carried out on the Zeiss Merlin or the Gemini 800 with in-lens detectors and an Oxford Instruments EBSD detector. The accelerating voltage was set to 30 kV and a 50-µm aperture was used. For EBSD, the sample was tilted at 70° towards the detector, approximately 17 mm from the scanning electron microscope pole piece, with the EBSD detector situated approximately 20–25 mm from the sample surface. The EBSD was calibrated for Cu_2_O, Au and Si patterns before the construction of each map to maximize the chance of successful identification and orientation measurement. All high-resolution cross-sectional transmission electron micrographs were collected with a Tecnai Osiris (FEI) microscope on electron-transparent samples prepared by focused ion beam sampling. Elemental composition was analysed by the Escalab 250Xi X-ray photoemission spectroscopy instrument. The bulk chemical analysis was carried out by combining X-ray photoelectron spectroscopy with argon ion gun etching with an ion energy of 1,000 eV.

### PEC measurements

All PEC measurements were implemented in a three-electrode configuration with Cu_2_O photocathodes as the working electrodes, platinum wires as the counter electrodes and Ag/AgCl reference electrodes. The pH 5.01 and pH 7.01 buffer electrolytes were prepared by tuning the 0.5 M Na_2_SO_4_, 0.1 M K_2_HPO_4_ and 0.1 M KH_2_PO_4_ phosphate solution. The pH 9.03 buffer was prepared by tuning the 0.5 M Na_2_SO_4_, 0.1 M Na_2_CO_3_ and 0.1 M NaHCO_3_ carbonate solution with sulfuric acid. Potentiostats (SP-200 or SP-150e; Biologic) were used to acquire the photoresponse under chopped illumination from the LCS-100 solar simulator (class ABB; Newport) with a built-in AM1.5 G filter. Calibration was carried out across the 300–800 nm wavelength region with a certified silicon diode behind a KG3 filter. All linear-sweep voltammetry was carried out at a 10 mV s^−1^ rate. Potentials versus the Ag/AgCl reference electrode were transformed to the reversible hydrogen electrode scale using the following equation:

$${E}_{{\rm{R}}{\rm{H}}{\rm{E}}}={E}_{{\rm{A}}{\rm{g}}/{\rm{A}}{\rm{g}}{\rm{C}}{\rm{l}}(3MKCL)}+0.197\,{\rm{V}}+0.059\,{\rm{V}}\times {\rm{p}}{\rm{H}}$$

Both stability tests and IPCEs were measured in the pH 5.0 buffer at 0.5 V versus the RHE. The IPCE was measured by comparing the wavelength-dependent photoresponse of the photoelectrodes with that of a silicon photodiode (FDS100-CAL, Thorlabs) using light from a 300-W xenon lamp passed through a monochromator (TLS300XU, Newport). Current readings were taken 5 s after each wavelength change. The PEC performance with sacrificial agent was obtained by scanning the photocathodes in 0.1 M europium(III) nitrate solution with simulated one-sun illumination. Details on calculation of the charge separation efficiencies are provided in Supplementary Discussion 4.

### Gas quantification

The faradaic efficiency of the photocathode was measured in a gas-tight PEC one-room cell. An Ag/AgCl (KCl saturated) reference electrode was used, and a platinum foil was used as the counter electrode. Epoxy was used to mask exposed parts of the cathode. The solution in the cell was vigorously stirred at 400 r.p.m. and constantly purged with argon at a rate of 13 sccm, controlled by a mass flow controller. The gas outlet was connected to a safe bottle that prevents the entry of water vapour into the gas chromatograph. These samples were subsequently analysed by gas chromatography (GC9790plus) every 20 min with 10 min analysing time and 10 min gap. The average exhaust gas flow rate was 12.4 sccm calculated by a burette. Gas chromatography system calibration and faradaic efficiency calculations are described in Supplementary Discussion 5.

### Measurements for electronic and optical properties of semiconductors

The electrochemical impedance measurements were carried out in the dark with a Biologic 150e potentiostat. The photocathodes were immersed in a 1 M sodium sulphate solution with a Ag/AgCl reference electrode and Pt wire to form a three-electrode configuration. The potential was varied in the stable region defined by the Pourbaix diagram with an a.c. amplitude of 10 mV superposed on the d.c. component. The space-charge capacitance of the semiconductor varied as a function of the applied potential according to the Mott–Schottky equation as shown below:

$$\frac{1}{{C}^{2}}=\frac{2}{\varepsilon {\varepsilon }_{0}e{N}_{{\rm{A}}}{A}^{2}}\left(-E+{E}_{{\rm{fb}}}-\frac{kT}{e}\right)$$

in which *C* is the interfacial capacitance, *A* is the electrode active area, *ε*_0_ is the vacuum permittivity, *ε* = 7.5 (ref. ^20^) is the relative dielectric constant, *E* is the applied potential, and *E*_fb_ is the flat band potential. The majority carrier concentrations (*N*_A_)—that is, the hole concentrations—in the Cu_2_O thin films of three orientations were estimated. *I–V* responses of the hole-only Cu_2_O devices were recorded using a Keithley 2450 SourceMeter with the assistance of a d.c. probe station. The Cu_2_O hole-only device was prepared by sandwiching the Cu_2_O layer with Au and MoO_x_/Ag layers. A 10-nm layer of MoO_*x*_ and 80 nm of Ag were evaporated onto the Cu_2_O to prevent short-circuiting and obtain a suitable workfunction. The samples were kept in a dark environment under a vacuum at room temperature during the measurement. The nonlinear responses with characteristic slopes (ohmic region, *n* = 1 and Child’s region, *n* = 2) were acquired and analysed according to the Mott–Gurney law:

$$j=\frac{9\varepsilon {\varepsilon }_{0}\,\mu {V}^{2}}{8{L}^{3}}$$

in which *V* is the applied bias, *ε*_0_ is the vacuum permittivity, *ε* (=7.5) is the relative dielectric constant and *L* is the thickness of the Cu_2_O layer (Supplementary Figs. 12–14 and Supplementary Tables 1 and 2). The voltages were determined by the trap densities (*n*_t_)

$${V}_{{\rm{TFL}}}=\frac{e{n}_{{\rm{t}}}{L}^{2}}{2\varepsilon {\varepsilon }_{0}}$$

in which *e* is the elementary charge and *L* is the sample thickness. The ultraviolet–visible reflectance spectra of Cu_2_O were collected on a Shimadzu UV-3600 Plus double-beam spectrophotometer in single-beam mode and using an ISR-603 Integrating Sphere Attachment (integrating sphere: 60 mm in diameter). For the measurement of total and specular reflectance spectra, the samples were placed in the respective position on the integrating sphere. The Au-sandwiched SC-Cu_2_O devices, for transfer length method measurements, were prepared by evaporating 100 nm of patterned Au layer on the Cu_2_O/Au/Si thin films. The single-crystal Au and evaporated Au were wired to a Keithley 2450 SourceMeter using Ag paste. *J–V* curves were recorded by scanning between −0.1 V to 0.1 V in the dark at room temperature. Owing to the interest in out-of-plane properties of the single-crystal films, the transfer length method measurement was modified. In contrast to the conventional transfer length method, the variable of length is replaced with thickness (five for each crystal orientation). By extrapolating to the intersection point on the y-axis, contact resistivity can be achieved. Photoluminescence measurements were carried out on an IMA Vis hyperspectral microscope. A 405-nm, continuous-wave laser was beam-shaped to form a top-hat profile and was focused onto the back focal plane of an Olympus ×100 air objective lens, producing a wide-field, flat illumination profile. The laser light excited the sample, producing photoluminescence that was collected by the same objective lens. A dichroic beamsplitter removed the excitation light before the light was guided onto a volume Bragg grating that spectrally split the light onto a Hamamatsu ORCA-Flash4.0 V3 digital CMOS camera. The spectra were acquired using 180 W cm^−2^ excitation with spectral calibration and dark subtraction.

### Transient reflection spectroscopy

Transient reflection spectroscopy measurements were carried out on two set-ups. In the first set-up (ultraviolet–visible probe), the output of a Ti:sapphire amplifier system (Spectra Physics Solstice Ace) operating at 1 kHz and generating pulses of about 100 fs was split into pump and probe beam paths. The 400-nm pump pulses were created by sending the 800-nm fundamental beam of the Solstice Ace through a second-harmonic-generating β-barium borate crystal (Eksma Optics). The pump was blocked by a chopper wheel rotating at 500 Hz. For the probe light path, a mechanical stage (Thorlabs DDS300-E/M) was used to adjust the delay between the pump and the probe. The ultraviolet–visible broadband beam (330–700 nm) was generated by focusing the 800-nm fundamental beam from the mechanical stage onto a CaF_2_ crystal (Eksma Optics, 5 mm) that was connected to a digital motion controller (Mercury C-863 DC Motor Controller). The reflected pulses were collected with a monochrome line scan camera (JAI SW-4000M-PMCL, spectrograph: Andor Shamrock SR-163) with collected data fed straight into the computer. In the second set-up (visible–near infrared probe), the output of a Ti:sapphire amplifier system (Spectra Physics Solstice Ace) operating at 1 kHz and generating pulses of about 100 fs was split into two beam paths (pump and probe). The 400-nm pump pulses were created by sending the 800-nm fundamental beam through a second-harmonic-generating β-barium borate crystal (Eksma Optics). The pump was blocked by a chopper wheel rotating at 500 Hz. The visible broadband beam (520–780 nm) was generated in a home-built noncollinear optical parametric amplifier and was sent to a computer-operated mechanical delay stage (Thorlabs DDS300-E/M) to adjust the pump–probe delay. The white light from the delay stage was split into two identical beams (probe and reference) by a 50/50 beamsplitter. The reference beam did not interact with the pump at the sample, which allows for correcting for any shot-to-shot fluctuations. The reflected probe and reference pulses were collected with a silicon dual-line array detector (Hamamatsu S8381-1024Q, spectrograph: Andor Shamrock SR-303i-B) driven and read out by a custom-built board (Stresing Entwicklungsbüro). Calculations for carrier diffusion length, pump fluence and carrier density are discussed in Supplementary Discussion 1.

## Online content

Any methods, additional references, Nature Portfolio reporting summaries, source data, extended data, supplementary information, acknowledgements, peer review information; details of author contributions and competing interests; and statements of data and code availability are available at 10.1038/s41586-024-07273-8.

## Supplementary information

Supplementary Information Supplementary Discussions 1–5, Tables 1–7, Figs 1–38 and References.

## Data Availability

The data that support the findings of this study are available from the University of Cambridge repository at 10.17863/CAM.107201.
