# Publisher Correction: High carrier mobility along the [111] orientation in Cu_2_O photoelectrodes

**DOI:** 10.1038/s41586-024-07489-8

**Published:** 2024-05-08

**Authors:** Linfeng Pan, Linjie Dai, Oliver J. Burton, Lu Chen, Virgil Andrei, Youcheng Zhang, Dan Ren, Jinshui Cheng, Linxiao Wu, Kyle Frohna, Anna Abfalterer, Terry Chien-Jen Yang, Wenzhe Niu, Meng Xia, Stephan Hofmann, Paul J. Dyson, Erwin Reisner, Henning Sirringhaus, Jingshan Luo, Anders Hagfeldt, Michael Grätzel, Samuel D. Stranks

**Affiliations:** 1 Department of Chemical Engineering and Biotechnology, University of Cambridge, Cambridge, UK; 2 Cavendish Laboratory, University of Cambridge, Cambridge, UK; 3 Laboratory of Photonics and Interfaces, Institute of Chemical Sciences and Engineering, École Polytechnique Fédérale de Lausanne, Lausanne, Switzerland; 4 Laboratory of Photomolecular Science, Institute of Chemical Sciences and Engineering, École Polytechnique Fédérale de Lausanne, Lausanne, Switzerland; 5 Department of Engineering, University of Cambridge, Cambridge, UK; 6 Laboratory of Organometallic and Medicinal Chemistry, Institute of Chemical Sciences and Engineering, École Polytechnique Fédérale de Lausanne, Lausanne, Switzerland; 7 Yusuf Hamied Department of Chemistry, University of Cambridge, Cambridge, UK; 8 Institute of Photoelectronic Thin Film Devices and Technology, State Key Laboratory of Photovoltaic Materials and Cells, Ministry of Education Engineering Research Centre of Thin Film Photoelectronic Technology, Renewable Energy Conversion and Storage Centre, Frontiers Science Center for New Organic Matter, Nankai University, Tianjin, China; 9 Department of Chemistry-Ångström Laboratory, Uppsala University, Uppsala, Sweden

**Keywords:** Devices for energy harvesting, Solar fuels

Correction to: *Nature* 10.1038/s41586-024-07273-8 Published online 24 April 2024

In the version of the article initially published, Linfeng Pan and Linjie Dai were not named as equal contributors. This has now been corrected in the HTML and PDF versions of the article.

