# Supplementary material for: High carrier mobility along the [111] orientation in Cu2O photoelectrodes
Source: Nature. 2024 Apr 24;628(8009):765–70. doi: 10.1038/s41586-024-07273-8 (PMC11043049; doi:10.1038/s41586-024-07273-8)
Supplement: Supplementary file 1 — Supplementary Discussions 1–5, Tables 1–7, Figs 1–38 and References. [file 41586_2024_7273_MOESM1_ESM.pdf]

nature portfolio

https://doi.org/10.1038/s41586-024-07273-8

Supplementary information

High carrier mobility along the [111] orientation in Cu_2O photoelectrodes

In the format provided by the authors and unedited

Supplementary Information for:

High carrier mobility along the [111] orientation in Cu_2O photoelectrodes

Supplementary Discussion 1 │Calculation of pump fluence and carrier density

The diffusion length is proportional to the square root of the product of mobility and carrier lifetime.

$$L = \sqrt{D\tau}$$

where D is the diffusion coefficient, τ is the lifetime (the longest component) measured by transient absorption. According to the Einstein–Smoluchowski diffusion equation, the diffusion coefficient can be calculated as

$$D = \mu \frac{k_{\mathrm{B}}T}{e}$$

where μ is the carrier mobility, k_B is the Boltzmann constant, T is the absolute temperature, and e is the elementary charge. Mobility is inversely proportional to the scattering rate and the effective mass,

$$\mu = \frac{e\tau_{\mathrm{c}}}{m^*}$$

where τ_c is the collision time, and m^* is the effective mass of the carriers. In the case of Cu_2O, the mobility ratio of majority carriers (holes) and minority carriers (electrons) holes is inversely proportional to their effective mass ratio, assuming a similar scattering rate for both carriers.

Fluence per pulse is calculated by Fluence (per pulse) = $P/(f\times S)$, where P is the power of the pump, which can be measured by a power meter, f = 500 Hz is the repetition rate of the TA system, $S = \pi r^2$ is the area of the pump beam spot (r: beam spot radius measured by a beam profiler). In our work, spot size (diameter) = $2 \times r$ = 1300 μm for 15-nm Cu_2O film measurements and $2 \times r$ = 1000 μm for 500-nm Cu_2O film measurements.

Carrier density is calculated by:

Carrier density (per pulse) = The number of photons absorbed (per pulse) / d, where d is the film thickness. The number of photons absorbed (per pulse) = Fluence (per pulse) × $(1-e^{-\alpha d})/h\nu$, where α is the absorption coefficient of Cu_2O, d is the thickness of the film, h is the Planck constant, and ν is the frequency of light. The frequency-dependent absorption coefficient (α) can be expressed in terms of the extinction coefficient (κ) as $\alpha = 4\pi\kappa/\lambda$, where λ is the wavelength of the light. According to the reference[1], κ (400 nm) = 1.17743 for Cu_2O semiconductors.

Supplementary Discussion 2 | Smooth surface of single-crystal films

Although the SC-Cu_2O (111) photocathode excels at solar water splitting due to its outstanding carrier transport properties, its PEC performance is still not comparable with the state-of-the-art polycrystalline ones (Supplementary Fig. 23)[2,3]. To address this discrepancy, our efforts were directed towards gaining an optical understanding of the issue. Notably, the samples' extremely smooth surfaces exhibit mirror-like characteristics across all crystal orientations (as illustrated in Supplementary Figure 24a-c). In Supplementary Fig. 24d, a pair of tweezers is placed above, resulting in a very sharp reflection. The total reflectance spectra were recorded using the UV-vis instrument with an integrating sphere for both single-crystal and polycrystalline thin films (Supplementary Fig. 25). The spectra show that roughly 35% of the incident light within the Cu_2O bandgap region is reflected by SC-Cu_2O samples, while all poly-crystalline samples show less than 10% reflection. The peak absorption reaches 98% for poly-Cu_2O (111) ranging between 400-475 nm (Supplementary Fig. 25b). In the detailed spectra where specular and diffuse reflectance are separated, we found that the specular component accounts for a significant proportion of the total, which is clear evidence of the smooth surface (Supplementary Fig. 26). The specular-to-diffuse reflectance ratio is also associated with the visual surface roughness of the single crystal films featuring high-to-low ratio (smooth-to-rough) sequence of (110), (111) and (100) as observed in the SEM images of Supplementary Fig. 2. It is also noticeable that substantial interference happens on all samples with shifting peak positions for different thicknesses (Supplementary Figs. 27). We also confirmed that the interference occurs only on the surface of the Cu_2O single crystal films by comparing the reflectance spectra of bare Au films with various orientations and thicknesses (Supplementary Fig. 28). In addition, the smooth surface can lead to increases in workload per area of catalyst compared to the nanowire or polycrystal Cu_2O photocathodes. All results suggest that the very smooth surface of the single crystal films is responsible for the inferior performance compared to their polycrystalline counterparts.

Supplementary Discussion 3 │ Cost Estimation of the Cu_2O Layer

We estimate the cost of the Cu_2O layer according to the preparation processes described in literature[2–9]. A bottom-up model is applied as it builds up on the smallest and most fundamental elements that formulate the total cost[10–12]. We note that this is a rough estimation with a focus on the dominant materials cost among five categories in the bottom-up cost model developed by the National Renewable Energy Laboratory[11,13]. Nevertheless, the estimated materials cost of the electrochemical methods (Route 2 & 3), i.e., the liquid phase epitaxy and the conventional electrodeposition, for Cu_2O is more than three orders of magnitude lower compared to the one prepared by thermal oxidation. The evaluation is carried out at a small scale using lab chemicals, as none of the materials/devices are commercially available to our best knowledge[14]. All raw materials are selected at the purity mentioned in their corresponding references, ensuring alignment with their notable efficiency. The other categories such as utility and depreciation and large-scale production will also be discussed. Further work will be required to provide more accurate cost estimates, including how the costs might scale with volume and when adopted with larger scale tools and processes.

Types, dimensions, and quantity of the Cu_2O photocathodes

In total, four types of the state-of-the-art Cu_2O photocathodes are compared[2–9]. Three types of flat devices by thermal oxidation[7–9] liquid phase epitaxy and electrodeposition[3–5] (including this work), and one nanowire device via annealing and electrodeposition[2,6]. We assume a small-scale pilot production line producing 100 pieces of **10*10 cm^2** Cu_2O thin films, resulting in a total area of **1 m^2**. Their thin film thicknesses vary according to the corresponding best-performing photocathodes, i.e., 75 μm for thermal oxidation (final Cu_2O thickness), 1 μm for electrodeposition (final Cu_2O thickness) and 1.5 μm for nanowires (thickness of starting material). For annealing the 10*10 cm^2 sample, a 4-inch inner diameter tube furnace is used with an empirical argon flow rate of 0.5 L per min.

Preparation routes

Route 1: Cu_2O thin film by thermal oxidation[7–9]. Starting from the high-purity Cu foil (99.99%), the substrates are annealed in a quartz tube furnace under argon protection. The temperature ramped up from room temperature to around 1010 °C and was maintained for 2 hours. Following air purging, another 2 hours of argon-protected annealing was employed. It then ended with a cooling to room temperature in argon. Total argon purging is around 10 hours. 5 samples are annealed simultaneously in a 4-inch tube furnace.

Route 2 & 3: Cu_2O thin film by liquid phase epitaxy (Route 2) and traditional electrodeposition (Route 3) [3–5]. The electrolytes (1 L) of Route 2 & 3 share similar composition of 7.98 g $CuSO_4$, 67.5 g lactic acid, 21.77 g K_2SO_4, 84.15 g KOH and 1 kg deionized water. We assume that a total of 7.04 g $CuSO_4$ is separated into 10 portions and added into the electrolyte every 10 electrodepositions. Samples are rinsed with 50 mL of deionized water.

Route 4: Nanowire Cu_2O[2,6]. The starting thin film is the sputtered Cu layer (1.5 μm). Then an anodization is carried out in a 3 M KOH solution to form $Cu(OH)_2$ nanowire. The transformation to Cu_2O is completed in an argon-protected tube furnace (for a total of 12 h). The electrodeposited layer uses the electrolyte as prepared in route 2. Each 1 L electrolyte is used to prepare 20 Cu_2O thin films. Samples are rinsed with 100 mL of deionized water in two times.

Cost of the Cu_2O layer

Based on the above-mentioned model and cost of raw materials in the table below, the sole materials costs of 1 m^2 of Cu_2O layer via Route 1, 2 and 3 are £15341.1, £13.59 and £113.94, respectively. Electrodeposition (Route 2 & 3) stands out as the most cost-effective option for fabricating the Cu_2O layer, while the cost ratio between Route 1 and Route 2/3 is more than three orders of magnitude. Similarly, due to the costly thermal annealing under argon, Route 3 is about 8.4 times more expensive than Route 1. We find that the precursors for Cu_2O in Route 1 and 3 are contributing substantially to the total cost. The cost of argon is, in fact, more than the overall materials cost in Route 2 & 3. The advantage of electrodeposition was manifested in large-scale production, where the electrolyte can be reused by quantified addition of the consumed precursors. In practice, electrodeposition has proven to be very compatible with manufacturing large-area devices, which is very expensive in high-temperature processes.

Utility, depreciation, and environmental impact

We note that this is a rough comparison of the materials cost of only the Cu_2O layer at lab scale. Device configurations can vary in various routes. Nevertheless, the cost difference will be more drastic when energy consumption and instrument costs are considered because electrodeposition is known for its scalability, high production rates and ease of automation (roll-to-roll)[15–20]. Furnaces with atmosphere control and sputtering machines are costly, resulting in huge costs in depreciation. The ambient conditions and high materials usage ratio of electrodeposition also generate a much lower environmental impact compared to the other methods.

Supplementary Discussion 4 │ Charge separation efficiencies

The standard AM 1.5 G solar spectrum (wavelength-dependent photon flux) was obtained from the National Renewable Energy Laboratory (NREL) solar spectral irradiance data. In order to calculate the max current density of Cu_2O with an optical bandgap of around 2.0 eV[3,21], photons are integrated considering 100% incident photon-to-current conversion efficiency (IPCE), representing j_{max} in the following equation[22,23]:

$$j_{ph} = j_{max} \cdot \eta_{abs} \cdot \eta_{sep} \cdot \eta_{cat}$$

where j_{ph} is the solar hydrogen evolution current density, j_{max} is the max photon flux that certain semiconductors can absorb based on the AM1.5G spectrum (Supplementary Fig. 34a), η_{abs} is the absorptance (i.e., the ratio of absorbed photon flux to the incident flux or light harvesting efficiency Supplementary Fig. 34b), η_{sep} is the charge carrier separation efficiency and η_{cat} is the electrocatalytic efficiency for hydrogen evolution reaction. The η_{abs}, is a wavelength-dependent variable which can be calculated from absorbance (A) using the following equation:

$$\eta_{abs} = 1 - 10^{-A}$$

or from total reflectance (R_t) with the equation below:

$$\eta_{abs} = 1 - R_t$$

The catalytic efficiency is quantified as the proportion of electrons that engage in water reduction to form hydrogen, out of the total electrons that reach the interface between the photoelectrode and surface electrocatalysts. When a highly efficient electron acceptor (Eu^{3+}) is used in the electrolyte as sacrificial agent for photocathodes[24], the electrocatalytic efficiency is assumed to be $\eta_{cat} = 1$. Using the photocurrent density measured with sacrificial agent, j_{ph}^*, the charge separation efficiency, η_{sep}, can be calculated:

$$\eta_{sep} = \frac{j_{ph}^*}{j_{max} \cdot \eta_{abs}}$$

The following equations show the relationship between charge separation efficiency, IPCE (also known as the external quantum efficiency, EQE) and absorbed photon-to-current efficiency (APCE)[25].

$$APCE = \frac{IPCE}{\eta_{abs}}$$

$$APCE = \eta_{sep} \cdot \eta_{cat}$$

$$IPCE = \eta_{abs} \cdot \eta_{sep} \cdot \eta_{cat}$$

Supplementary Discussion 5 │ GC calibration and faradaic efficiency calculations

Calibration of the system was achieved by replacing the working electrode with a platinum foil (Tianjin Ai li) and running galvanostatic electrolyzes at 0.2, 0.5, 1, 1.5, 2, 3 mA using an electrochemical workstation (CHI-760E) generate a calibration curve (six points including the origin, linear fit R2 = 0.99893). The Cu_2O photocathode was tested at 0.5 V (versus RHE) under simulated AM 1.5G illumination (100 mW cm^{-2}) from an Xe-lamp (MC-X301B) equipped with an AM 1.5G filter. The intensity was controlled by light path distance which is determined by measuring the short-circuit current of a calibrated silicon diode with KG 3 filter.

The parameter table for calibration

Current (mA)	Exhaust gas flow rate (sccm)	charge (C)	Theoretical number of electron (μmol)	Theoretical hydrogen production (μmol)	Actual peak area
**0.2**	**13.0**	**0.0018**	**0.0191**	**0.0096**	**4567.725**
0.5	12.6	0.0048	0.0493	0.0247	11476.2
1	13.0	0.0092	0.0955	0.0478	22453
1.5	12.7	0.0142	0.1473	0.0737	35836.54
2	13.2	0.0182	0.1885	0.0943	47505
3	12.6	0.0285	0.2959	0.1479	71599.225

The parameters table for Cu_2O photocathodes test

Illumination time(min)	Peak area	Actual hydrogen production (μmol)	Current (mA)	Exhaust gas flow rate (sccm)	charge(C)	Theoretical number of electrons (μmol)	Theoretical hydrogen production (μmol)	FE (%)
**120**	**39918.2**	**0.0819**	**1.6**	**12.4**	**0.01552**	**0.161**	**0.0806**	**101.6**
140	40917.4	0.0839	1.55	12.4	0.015035	0.156	0.0780	107.5
160	42413.2	0.0870	1.53	12.4	0.014841	0.154	0.0770	112.9
180	39295.8	0.0806	1.5	12.4	0.01455	0.151	0.0755	106.7
200	38667.8	0.0793	1.43	12.4	0.013871	0.144	0.0720	110.2

System calibration

The volume of the sample loop (v_0) for H_2 in our gas chromatograph is 2 cm^3 and the exhaust gas flow rate of the gas is v = 13 cm^3 min^{-1} (the bold line). The time it takes to fill the sample loop is:

$$t = \frac{v_0}{v} = \frac{2\ cm^3}{13\ cm^3/min} = 0.154\ min = 9.2\ s$$

Theoretical number of electron (μmol) is denoted as N_{total}

$$N_{total} = \frac{I_0 \times t}{e} = \frac{\frac{0.2}{1000A} \times 9.2\ s}{1.602 \times 10^{-19}\ C/e} = 1.148 \times 10^{16} e = \frac{1.148 \times 10^{16}}{6.02 \times 10^{23}}\ mol$$
$$= 1.91 \times 10^{-8}\ mol = 0.0191\ \mu mol$$

The number of electrons required to form 1 molecule of H_2 is 2. So, the amount of hydrogen production ($n_{hydrogen}$.) can be calculated by

$$n_{theoretical\ hydrogen} = N_{total}/2 = 0.00955\mu mol$$

The peak area can be obtained from GC. After calculating the theoretical hydrogen at 0.2, 0.5, 1, 1.5, 2, 3 mA, respectively, we fit the peak area and theoretical hydrogen gas in origin, and the slope is 0.00000203979, and intercept is 0.000466857.

$$n_{hydrogen} = Area * 0.00000203979 + 0.000466857$$

Faradaic efficiency calculation for Cu_2O photocathodes

When the exhaust gas flow rate is 12.4 sccm, the time it takes to fill the sample loop is:

$$t = \frac{v_0}{v} = \frac{2cm^3}{12.4cm^3/min} = 0.16min = 9.7s$$

1 mol = 6.02*10^{23}

$$N_{total} = \frac{I_0 \times t}{e} = \frac{1.6/1000A \times 9.7s}{1.602 \times 10^{-19}C/e} = 9.688 \times 10^{16}e = \frac{9.688 \times 10^{16}}{6.02 \times 10^{23}}mol$$
$$= 1.61 \times 10^{-7}\ mol = 0.161\ \mu mol$$

$$n_{theoretical\ hydrogen} = \frac{N_{total}}{2} = 0.0806\ \mu mol$$

$$n_{actual\ hydrogen} = 39918.2 \times 0.00000203979 + 0.000466857 = 0.0819\ \mu mol$$

$$FE = \frac{n_{actual\ hydrogen}}{n_{theoretical\ hydrogen}} = \frac{0.0819\ \mu mol}{0.0806\ \mu mol} = 102\%$$

Supplementary Table 1 │ Thicknesses and growth rate calculation of thin SC-Cu_2O films. Average thicknesses were calculated using 10 thicknesses acquired on SEM images at two sites on each film. All films were grown by electrochemical epitaxy for 50 min.

Crystal orientation	Electrochemical epitaxial duration (min)	Average thickness (nm)	Growth rate (nm/min)
100	50	221.09	4.42
110	50	228.8	4.58
111	50	226.5	4.53

Supplementary Table 2 │ Thicknesses and growth rate calculation of thick SC-Cu_2O films. Average thicknesses were calculated using 10 thicknesses acquired on SEM images at two sites on each film. (100) and (110) films were grown by electrochemical epitaxy for 180 min and (111) for 230 min.

Crystal orientation	Electrochemical epitaxial duration (min)	Average thickness (nm)	Growth rate (nm/min)
(100)	180	800	4.44
(110)	180	810	4.5
(111)	230	1023.4	4.45

Supplementary Table 3 │ Carrier densities (cm^{-3}) calculated from the Mott-Schottky plots for SC-Cu_2O with various crystal orientations and frequencies.

Frequency	SC-Cu_2O (100)<br>area: 0.467 cm^2	SC-Cu_2O (110)<br>area: 0.591 cm^2	SC-Cu_2O (111)<br>area: 0.509 cm^2
2.511 kHz	3.866×10^{17}	7.82×10^{17}	7.25×10^{17}
1.586 kHz	4.63×10^{17}	7.83×10^{17}	8.32×10^{17}
1.000 kHz	5.57×10^{17}	7.85×10^{17}	8.87×10^{17}
631 Hz	6.29×10^{17}	8.15×10^{17}	9.15×10^{17}
398 Hz	6.69×10^{17}	8.37×10^{17}	9.94×10^{17}

Supplementary Table 4 │ Summary of orientation-dependent carrier properties acquired from the space charge limited current (SCLC) measurements. μ – carrier (hole) mobility; ρ – resistivity.

Crystal orientation	μ (cm^2 V^{-1} s^{-1})	ρ (Ω cm)	Carrier (hole) concentration (cm^{-3})	Trap density (cm^{-3})
(100)	1.29	2.45×10^3	1.91×10^{15}	8.22×10^{15}
(110)	0.87	2.68×10^3	2.72×10^{15}	8.88×10^{15}
(111)	15.4	4.7×10^2	1.81×10^{15}	2.18×10^{15}

Supplementary Table 5 │ Materials cost for the synthesis of Cu_2O layer via route 1.

Materials	Price (£/product)	Specifications	Producer	Cost (£/10 films)	Cost (£/m^3 film)	Remarks
Cu foil	204	thickness 0.1 mm, 10*10 cm^2, purity 99.999%	Thermo Scientific Chemicals	1530	15300	Calculation based on the used 75 μm considering no waste of materials
Argon	66.45	99.998%, 200 Bar, zero grade	Air Liquide	4.11	41.1	total volume 9.7 m^3

Supplementary Table 6 │ Materials cost for the synthesis of Cu_2O layer via route 2 & 3.

Materials	Price (£/product)	Specifications	Producer	Cost (£/10 films)	Cost (£/m^3 film)	Remarks
$CuSO_4$	190	≥99%, 500 g	Sigma Aldrich	3.03	5.70	electrolyte reused by addition of Cu_2SO_4
Lactic acid	163	~90%, 2.5 L, 1.2 g/mL	Sigma Aldrich	3.67	3.67	
K_2HPO_4	215	anhydrous, ≥98%, 2.5 kg	Sigma Aldrich	1.87	1.87	
KOH	305	≥85%, pellets, 12 kg	Sigma Aldrich	2.14	2.14	
Deionized water	around 0.035/L[26]			0.053	0.21	

Supplementary Table 7 │ Materials cost for the synthesis of Cu_2O layer via route 4.

Materials	Price (£/product)	Specifications	Producer	Cost (£/10 films)	Cost (£/m^3 film)	Remarks
$CuSO_4$	190	≥99%, 500 g	Sigma Aldrich	3.03	3.831	1 L minimum electrolyte for 10*10 cm^2 thin film, 300 nm Cu_2O electrodeposited
Lactic acid	163	~90%, 2.5 L	Sigma Aldrich	3.67	3.67	
K_2HPO_4	215	anhydrous, ≥98%, 2.5 kg	Sigma Aldrich	1.87	1.87	
KOH	305	≥85%, pellets, 12 kg	Sigma Aldrich	5.35	5.35	3 M KOH for anodization and 2 M KOH for electrodeposition
Deionized water	around 0.035/L[26]			0.105	0.42	
Cu sputtering target	421	76.2 mm*3.18 mm, 99.999%	Thermo Scientific Chemicals	4.95	49.5	Estimated usable area ratio: 53.47%, usable thickness of target: 75%
Argon	66.45	99.998%, 200 Bar, zero grade	Air Liquide	4.93	49.3	total volume 9.7 m^3

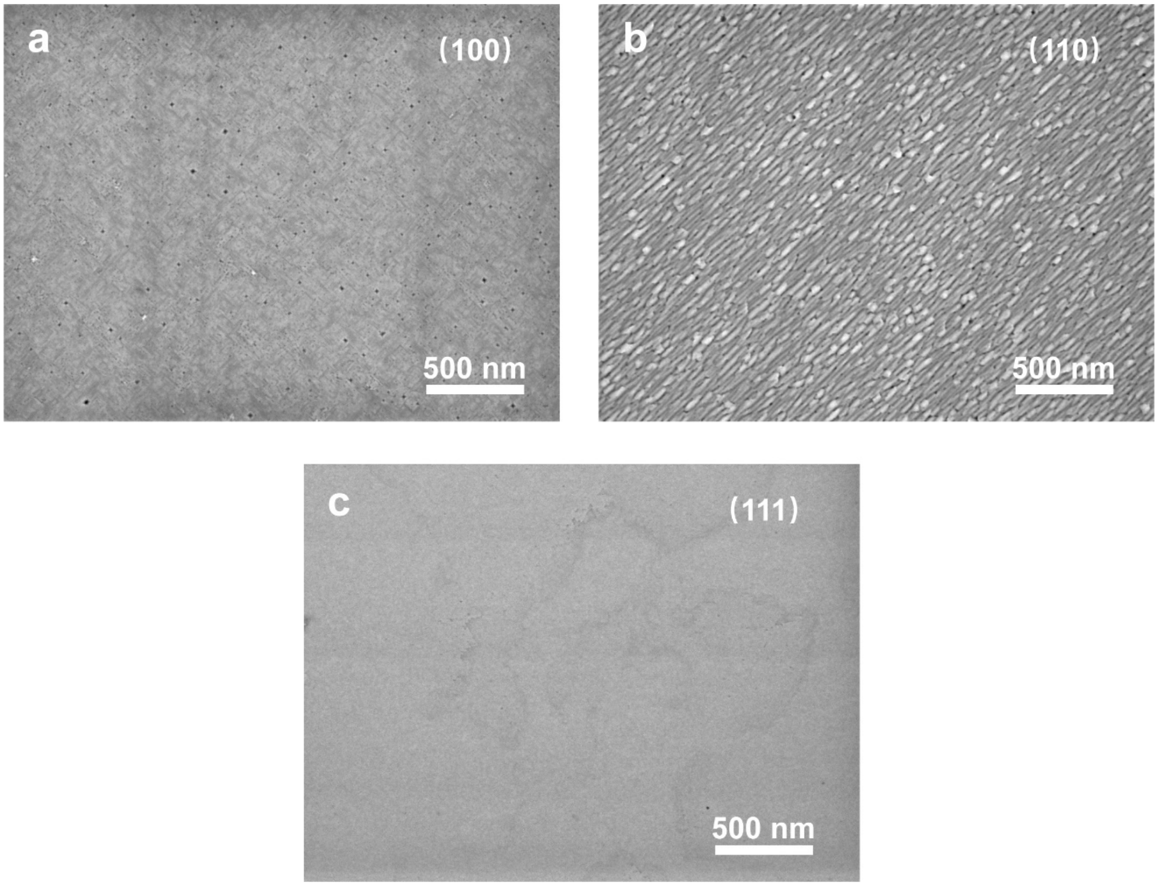

Supplementary Fig. 1 │Top-view scanning electron microscopy (SEM) images. a-c, SEM images of the epitaxial Au (100) (**a**), Au (110) (**b**) and Au (111) (**c**) layer on Si substrates with various crystal orientations.

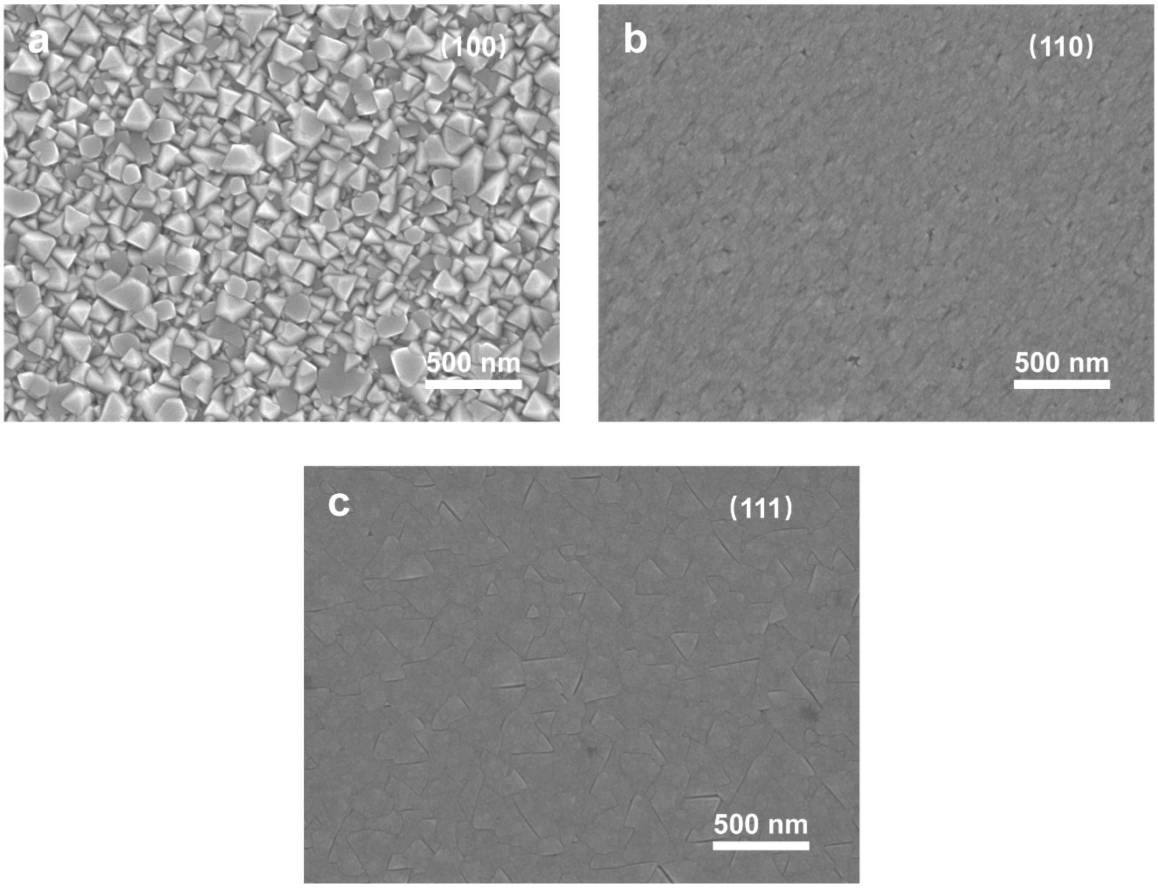

Supplementary Fig. 2 │Top-view scanning electron microscopy (SEM) images. a-c, SEM images of the epitaxial SC-Cu_2O (100) (**a**), SC-Cu_2O (110) (**b**) and SC-Cu_2O (111) layer (**c**) on Si substrates with various crystal orientations.

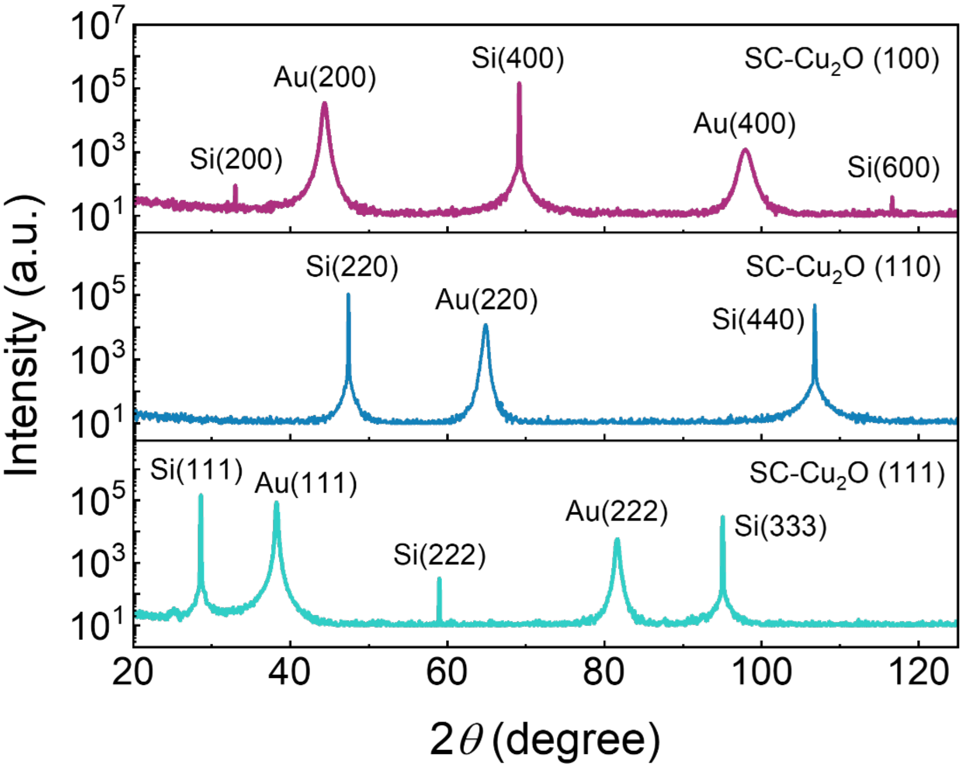

Supplementary Fig. 3 | X-ray diffraction patterns for the Au epitaxial layers on Si substrates with various crystal orientations.

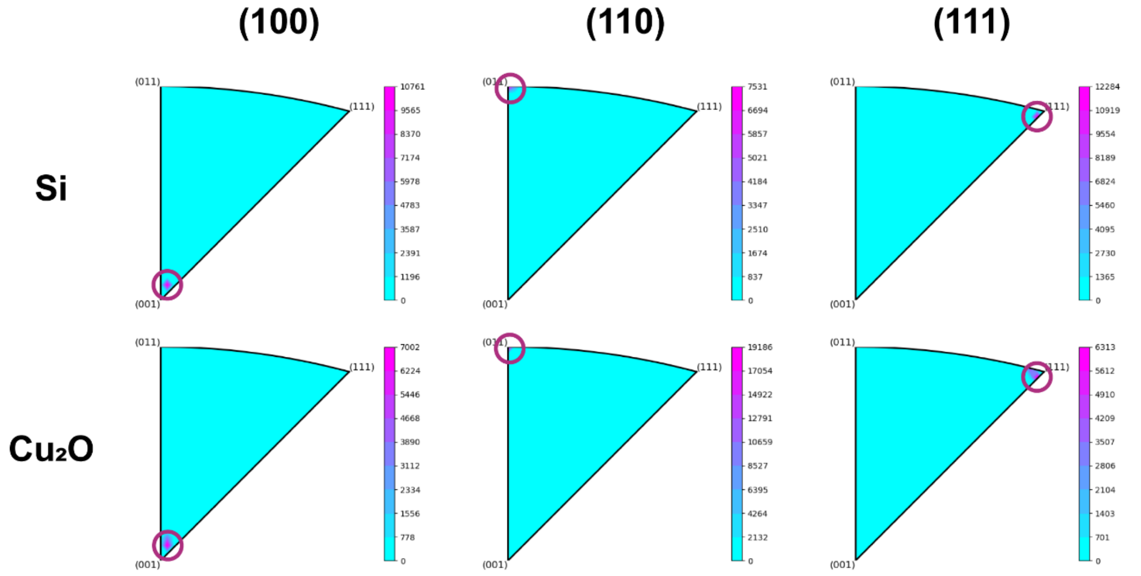

Supplementary Fig. 4 │ Inverse pole figures of SC-Cu_2O and Si substrates with various crystal orientations with circular marks highlighting the Si and Cu_2O reflections.

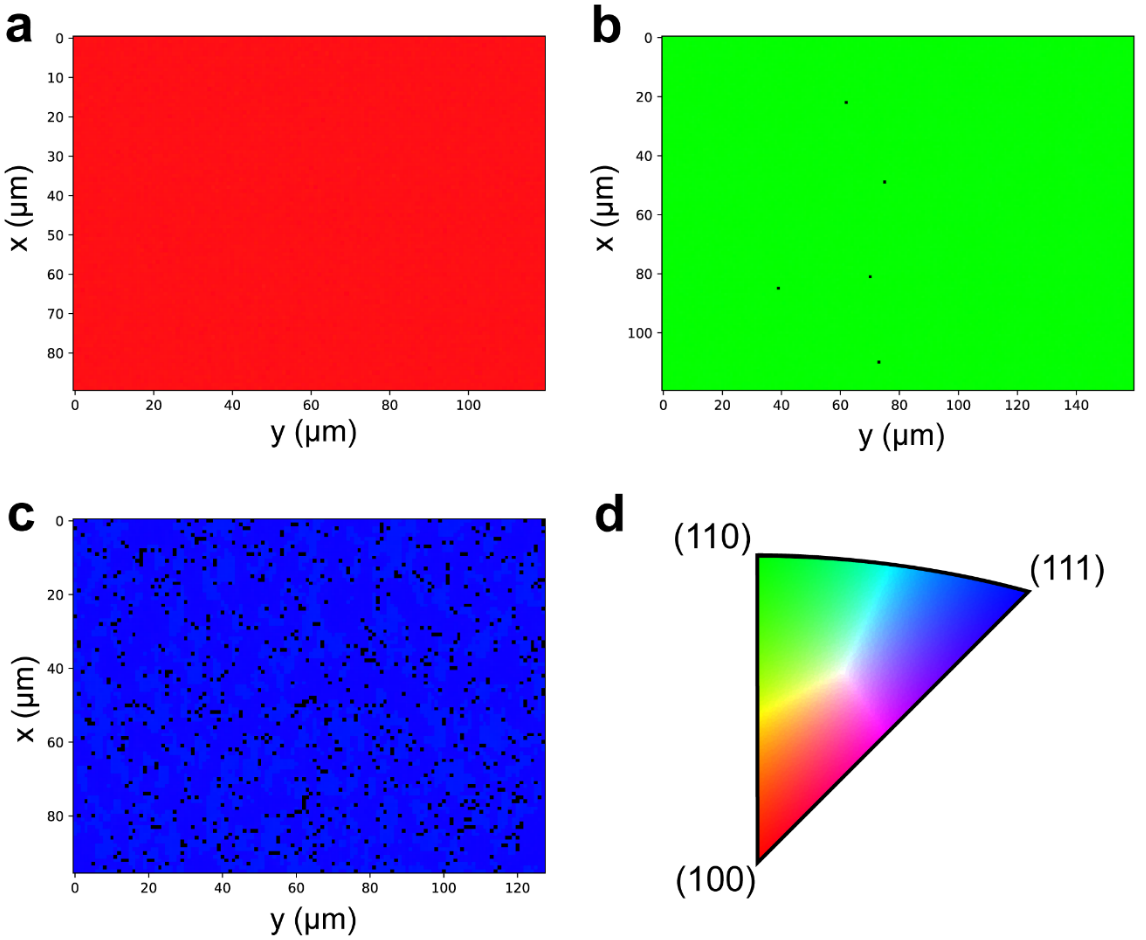

Supplementary Fig. 5 │ EBSD z-IPF maps of SC-Cu_2O thin films. a-c, z-IPF maps showing pure crystal orientations of (100) (**a**), (110) (**b**) and (111) (**c**) in single-crystal Cu_2O thin films. **d,** The Colour indicator for crystal orientations.

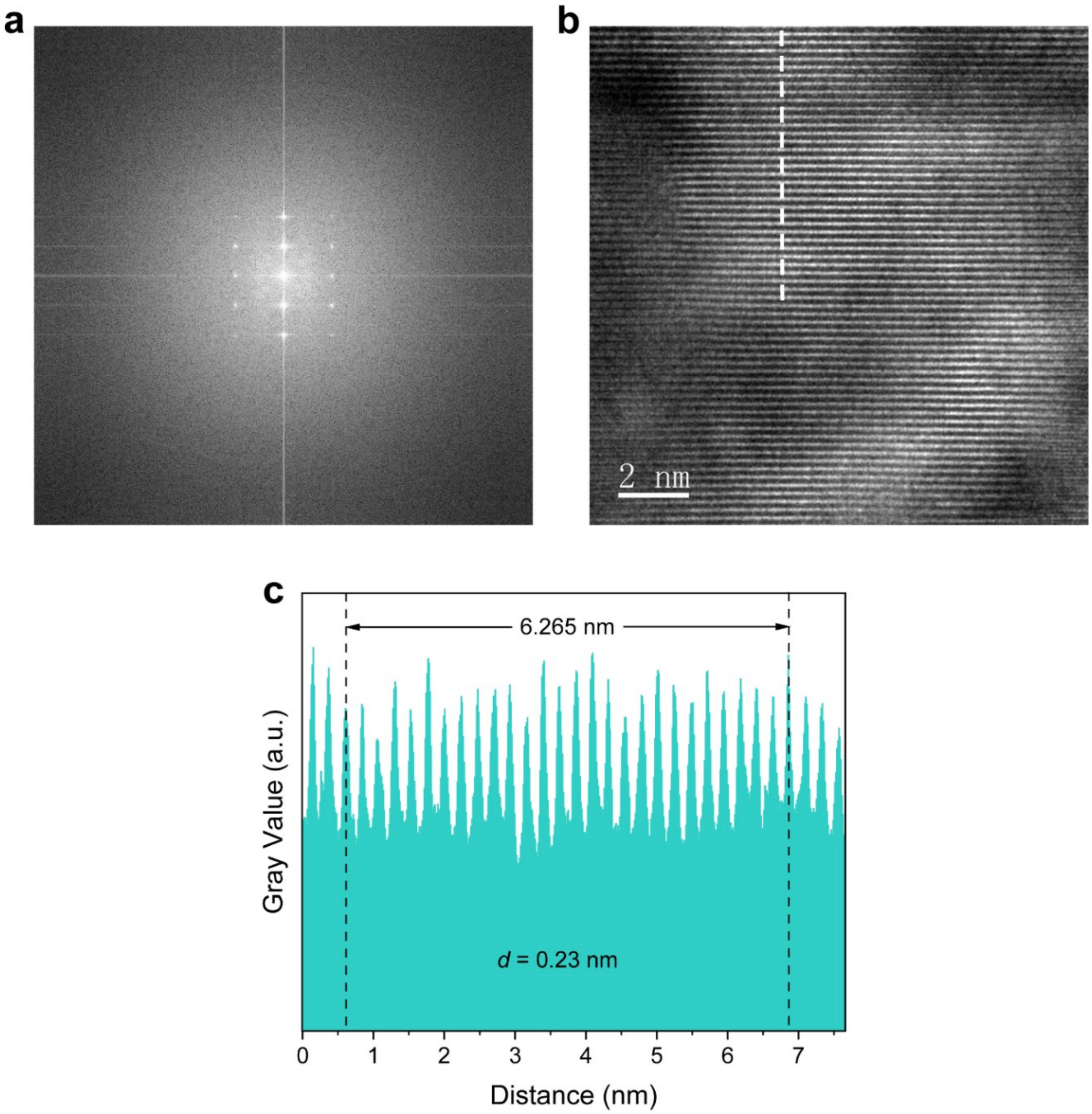

Supplementary Fig. 6 │Lattice spacing measurement in the Si layer. a, The fast Fourier transform image of a selected Si area in Fig. 1d. **b,** The corresponding filtered inverse fast Fourier transform (IFFT) image. **c,** The Intensity profile of the dashed line in **b**. Lattice spacing was calculated by measuring the distance between 27 peaks.

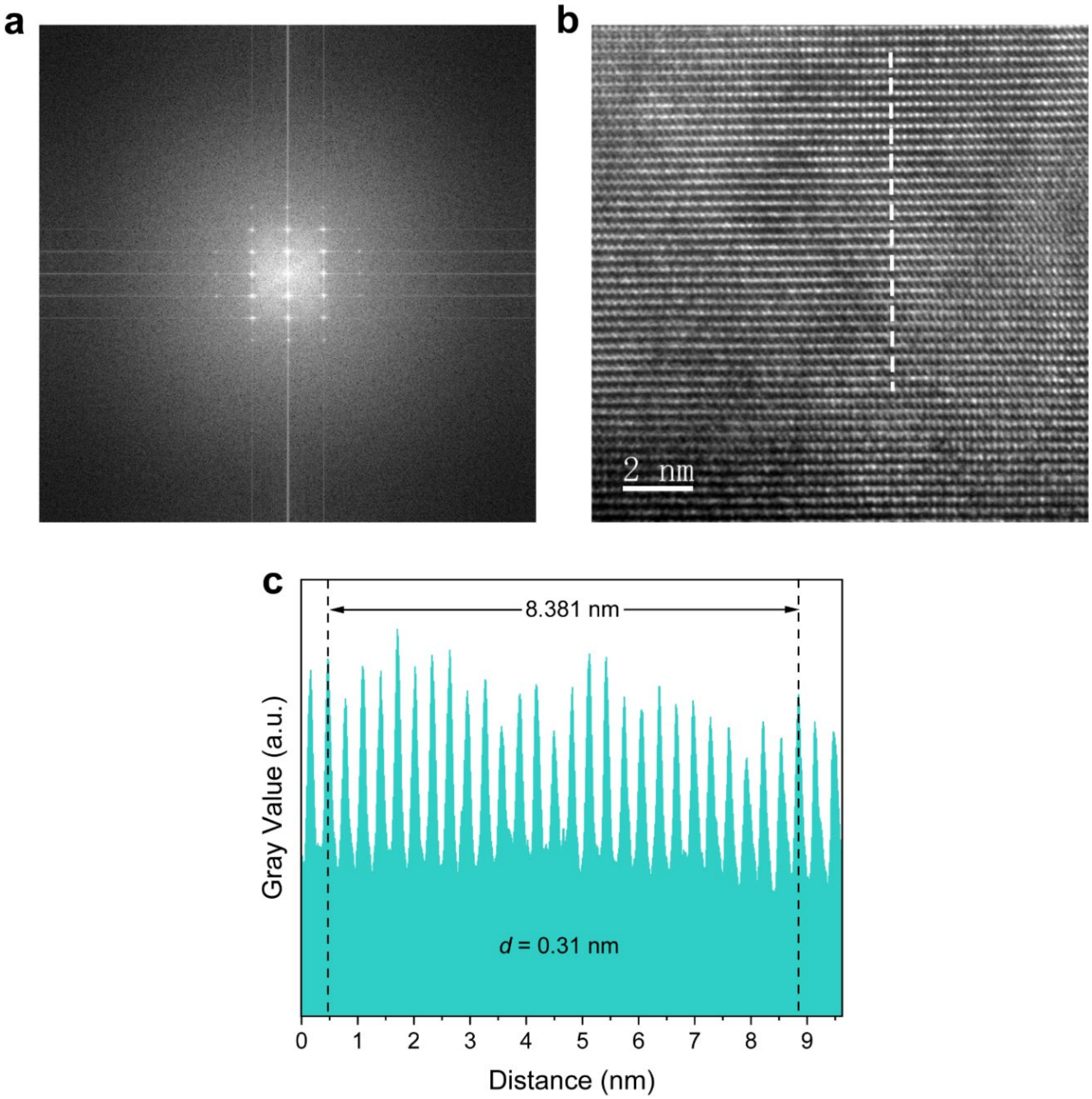

Supplementary Fig. 7 │Lattice spacing measurement in the Au layer. a, The fast Fourier transform image of a selected Au area in Fig. 1d. **b,** The corresponding filtered inverse fast Fourier transform (IFFT) image. **c,** The Intensity profile of the dashed line in **b**. Lattice spacing was calculated by measuring the distance between 27 peaks.

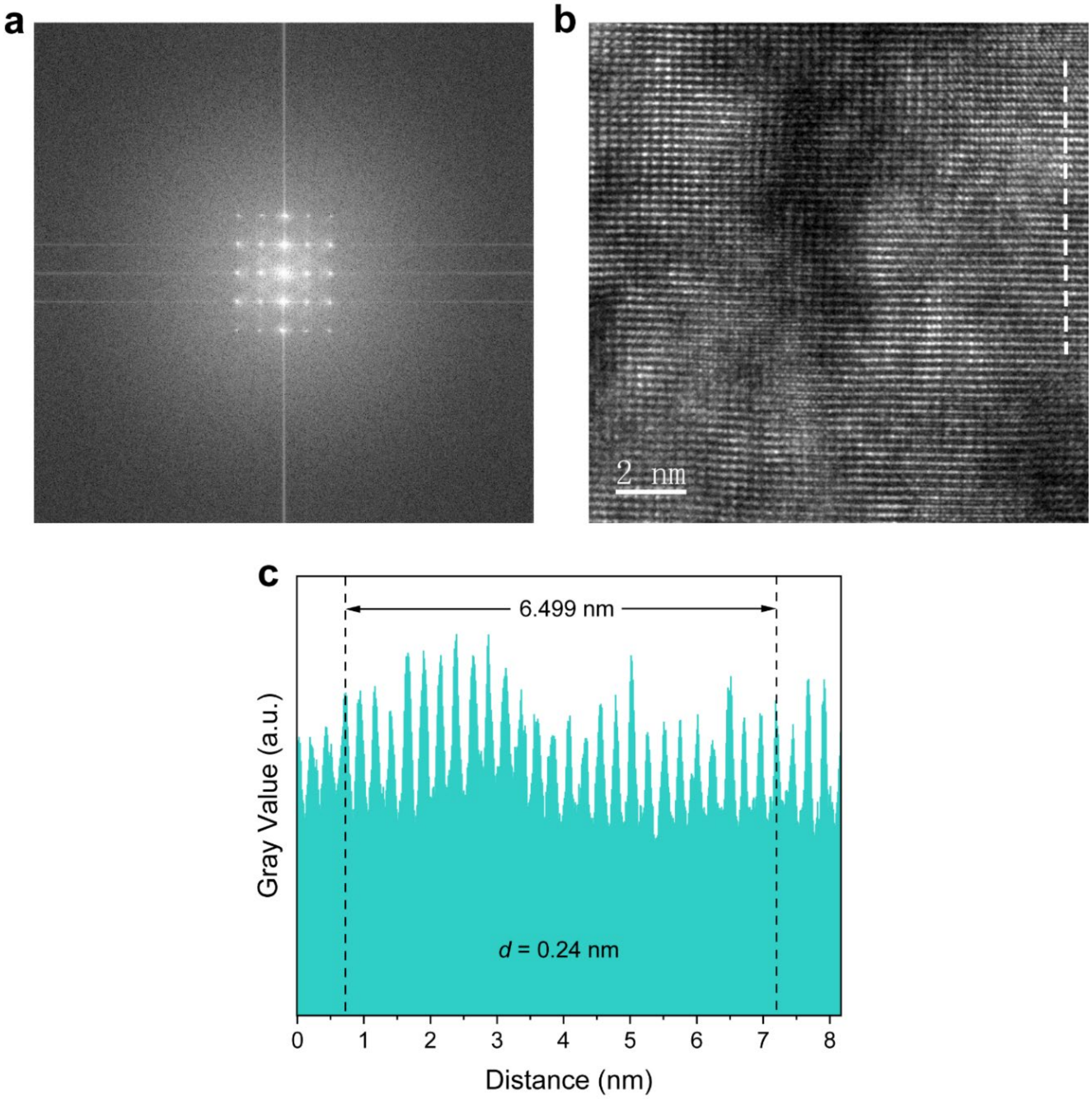

Supplementary Fig. 8 │Lattice spacing measurement in the Cu_2O layer. a, The fast Fourier transform image of a selected Cu_2O area in Fig. 1d. **b,** The corresponding filtered inverse fast Fourier transform (IFFT) image. **c,** The Intensity profile of the dashed line in **b**. Lattice spacing was calculated by measuring the distance between 27 peaks.

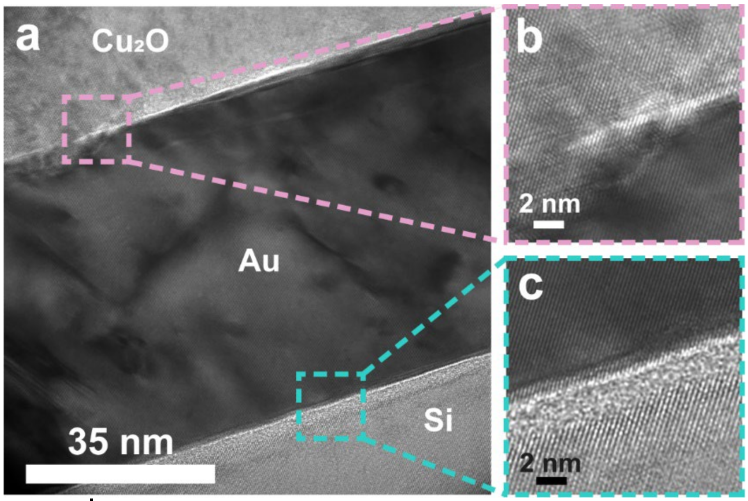

Supplementary Fig. 9 │ Cross-section TEM images. a-c, Cross-section TEM images showing Cu_2O (100) / Au (100) / Si (100) layers with close-up TEM images at the Cu_2O/Au interface (b) and the Au/Si interface (c).

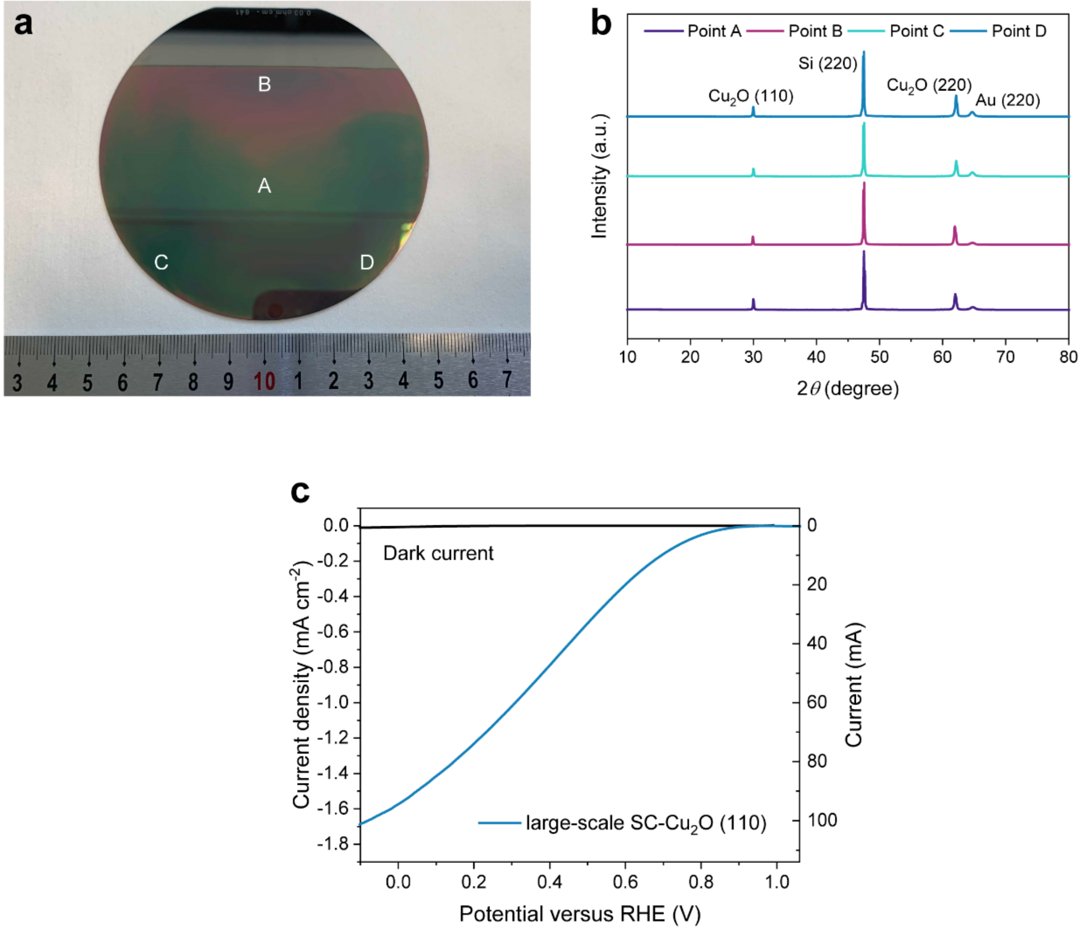

Supplementary Fig. 10 │ Large-scale demonstration of SC-Cu_2O (110) photocathode. a, Wafer-size single-crystal Cu_2O (110) thin film (active area: 60 cm^2). **b**, XRD patterns measured at the marked positions in **a** showing (110) single crystal orientation. **c**, Photoelectrochemical responses of wafer-size SC-Cu_2O photocathodes for solar hydrogen evolution under simulated one-sun condition.

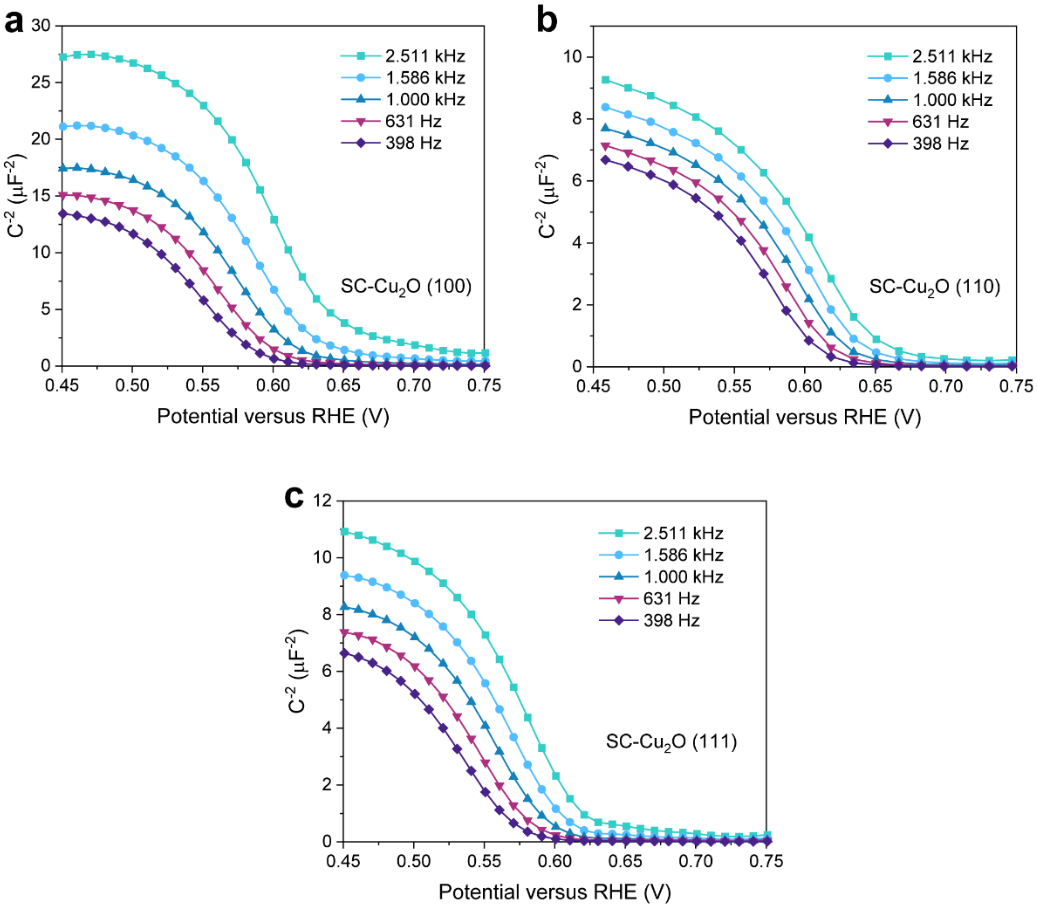

Supplementary Fig. 11 │ Mott-Schottky plots of SC-Cu_2O photocathode. a-c, Mott-Schottky plots of SC-Cu_2O (100) (**a**), (110) (**b**) and (111) (**c**) measured with 5 frequencies.

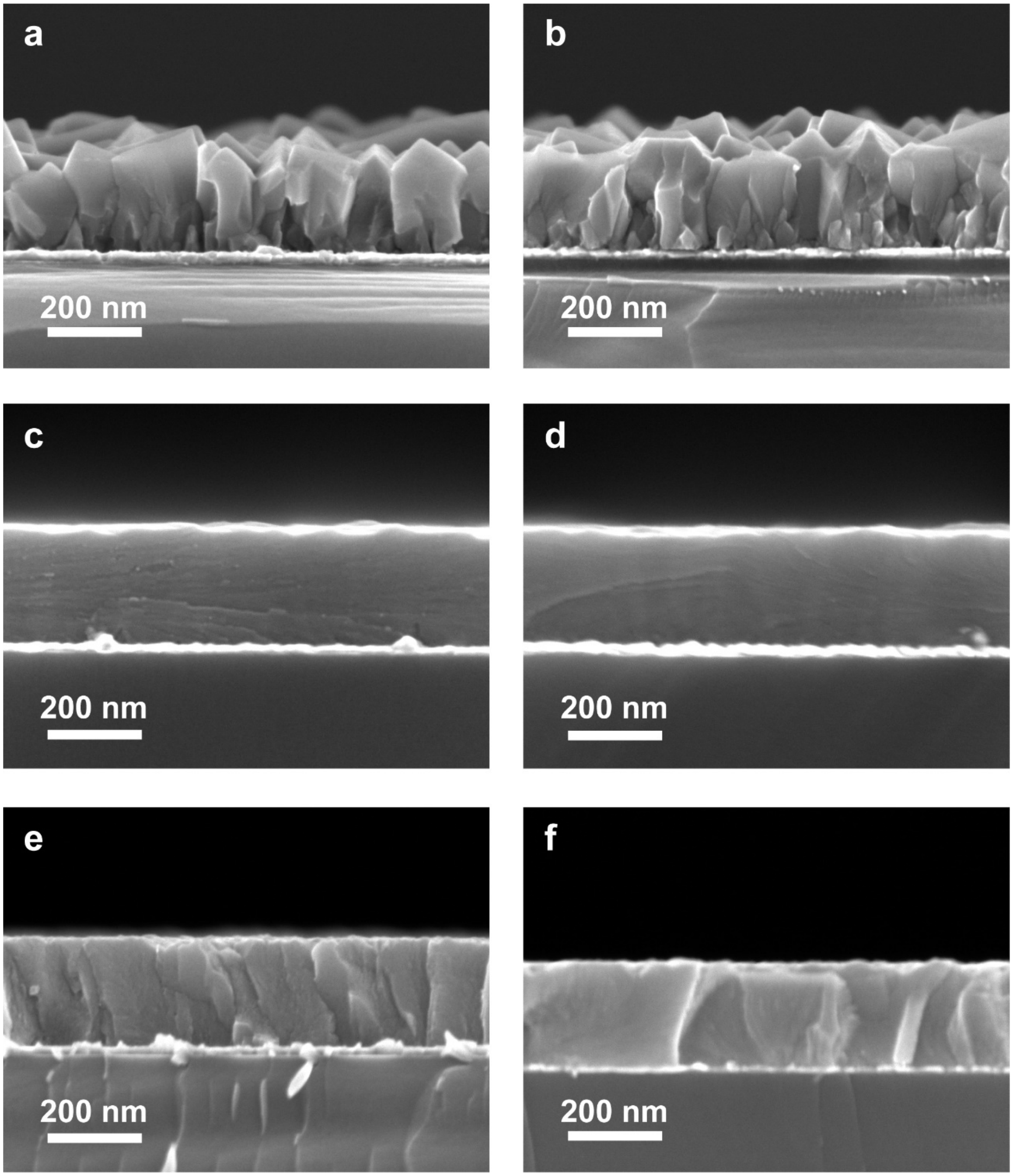

Supplementary Fig. 12 │Cross-section SEM images of thin SC-Cu_2O thin films. a-f, Cross-section SEM images of Cu_2O thin films of (100) (**a, b**), (110) (**c, d**) and (111) (**e, f**), which were grown by electrochemical epitaxy for 50 min.

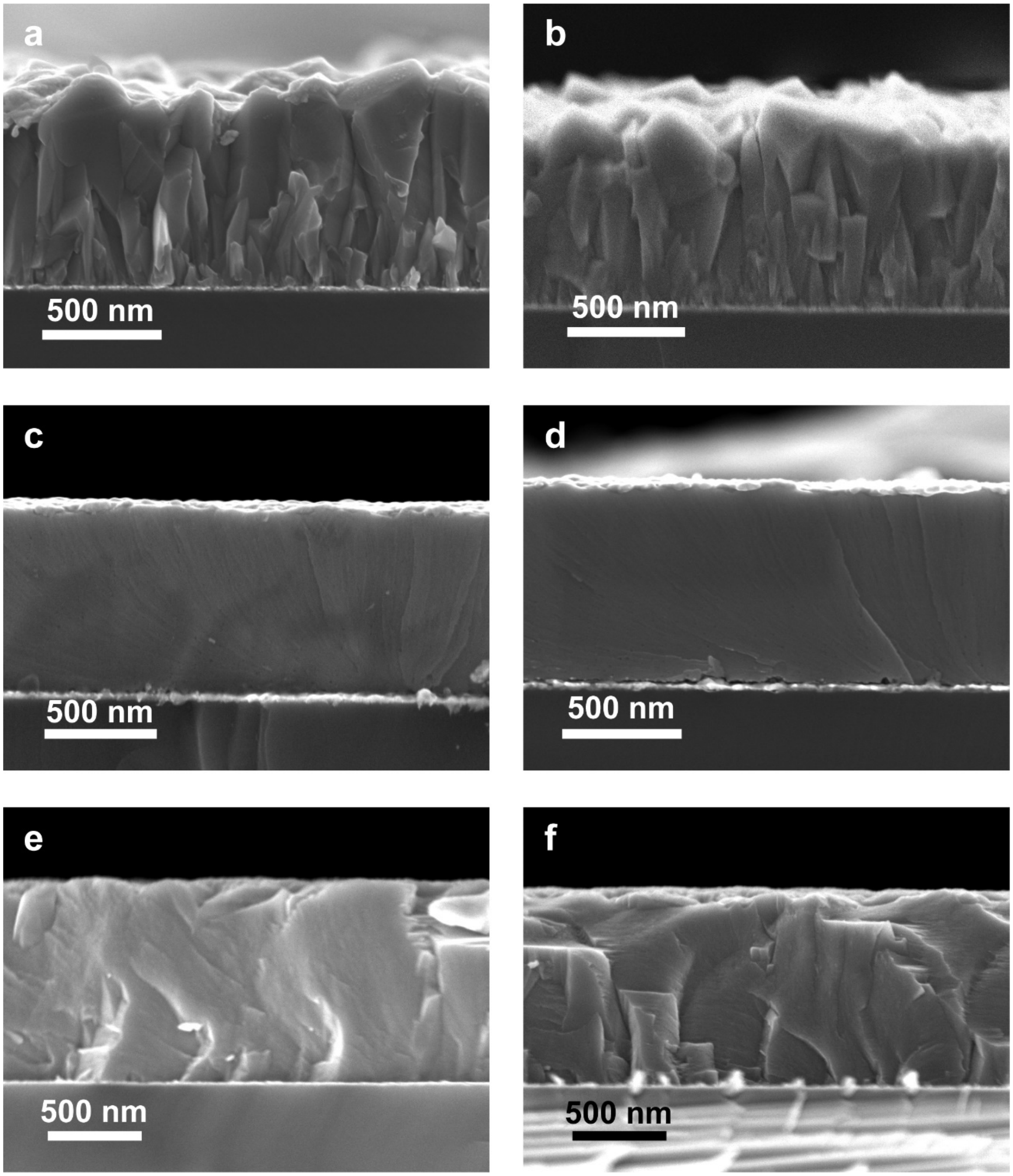

Supplementary Fig. 13 │ Cross-section SEM images of thick SC-Cu_2O thin films. a-f, Cross-section SEM images of the Cu_2O thin films of (100) (**a, b**) and (110) (**c, d**), which were grown by electrochemical epitaxy for 180 min. The Cu_2O thin films of (111) (**e, f**) were grown by electrochemical epitaxy for 230 min.

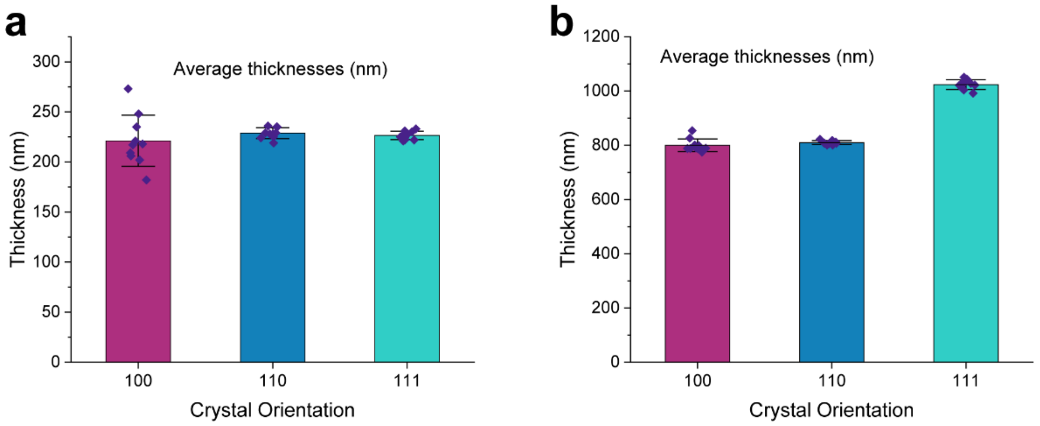

Supplementary Fig. 14 | Average thicknesses of the as-grown SC-Cu_2O thin films. All averaged thicknesses were determined using 10 thicknesses measured on SEM images acquired at two sites on thin SC-Cu_2O (**a**) and thick SC-Cu_2O films (**b**). The bar and error bar denote average thickness and the standard deviation, respectively.

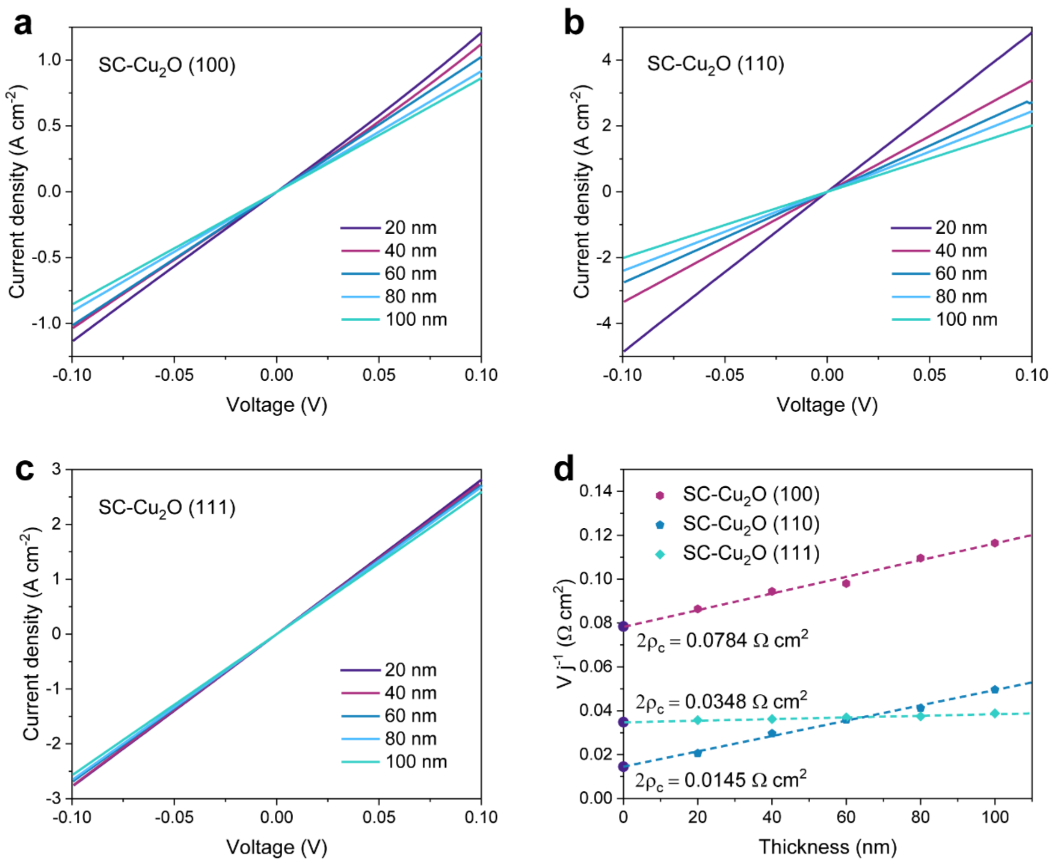

Supplementary Fig. 15 │ Contact resistance measurement between Au and Cu_2O via TLM. **a-c**, *j-V* curves of Au sandwiched single-crystal Cu_2O of (100) (**a**), (110) (**b**) and (111) (**c**) crystal orientations. **d**, $V j^{-1}$-t plots and linear fitting for single-crystal Cu_2O thin films.

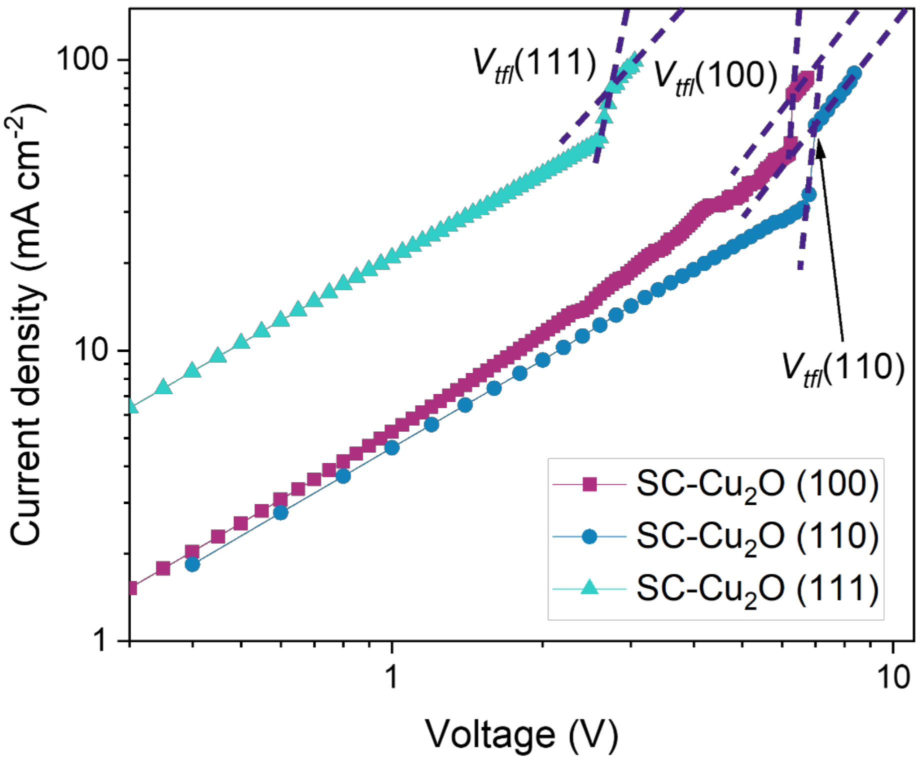

Supplementary Fig. 16 │ Comparison of current density-voltage curves measured on hole-only devices of SC-Cu_2O (100), (110) and (111).

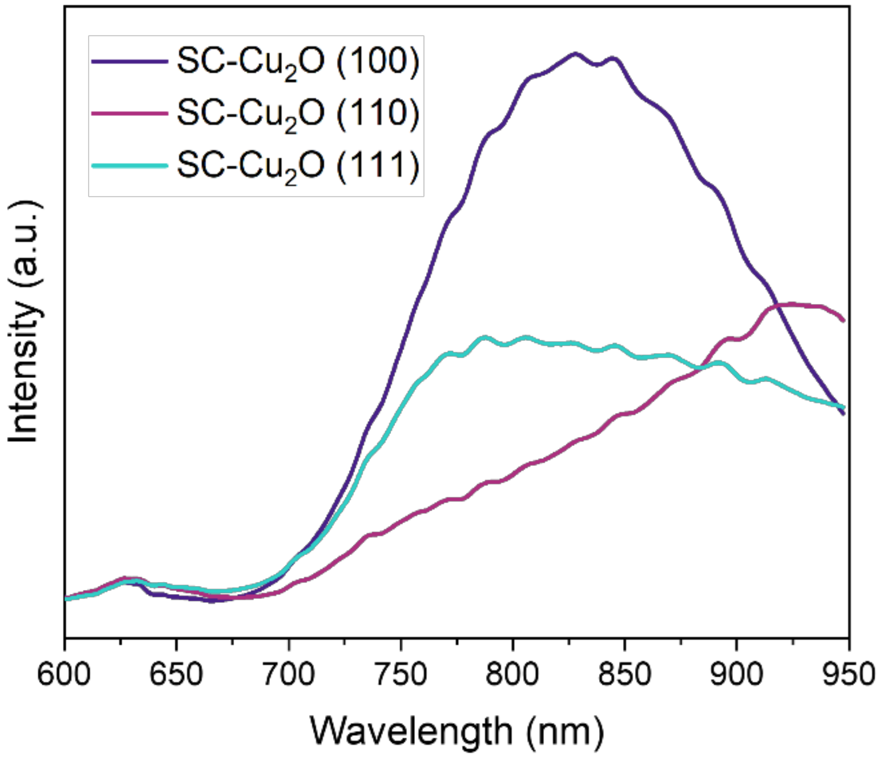

Supplementary Fig. 17 │ Photoluminescence (PL) spectra. Photoluminescence spectra of SC-Cu_2O thin films excited using a 405 nm continuous wave laser.

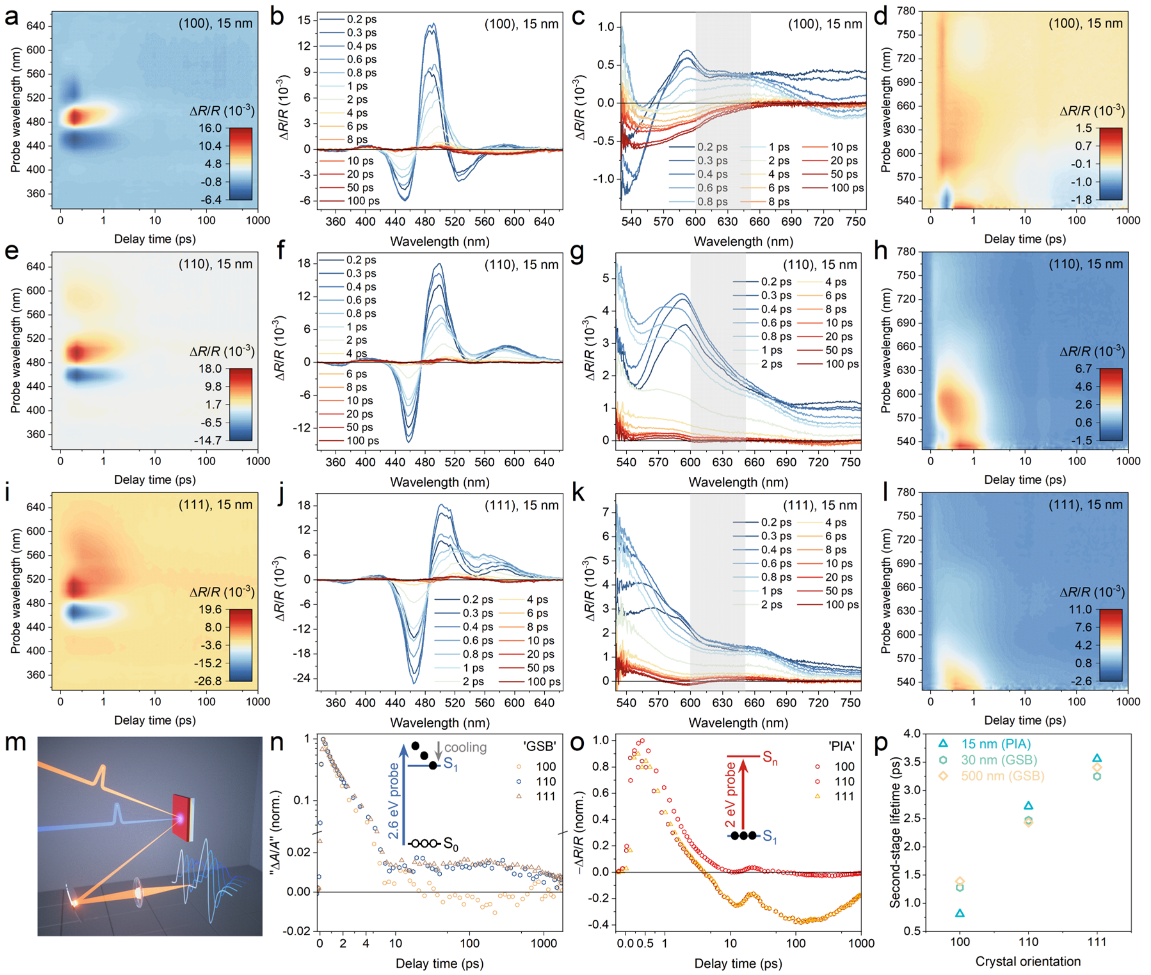

Supplementary Fig. 18 │ Transient reflection spectroscopy on SC-Cu_2O films with all crystal orientations. a-l, TR maps and corresponding TR spectra of 15-nm Cu_2O films with crystal orientations of (100) (**a-d**), (110) (**e-h**), and (111) (**i-l**) (pump wavelength, 400 nm, fluence, 110 μJ·cm^{-2}). **m,** Geometry of the applied transient reflection spectroscopy. **n, o**, Kinetics of the orientation-dependent photo-induced ground state bleach signals from the reproduced TA spectra (**n**), and the photo-induced absorption (600 – 650 nm) from the TR spectra (**o**) of SC-Cu_2O (extraction spectral ranges are indicated with a grey background in **c**, **g** and **k**). **p,** Second-stage lifetime of SC-Cu_2O films with various thicknesses.

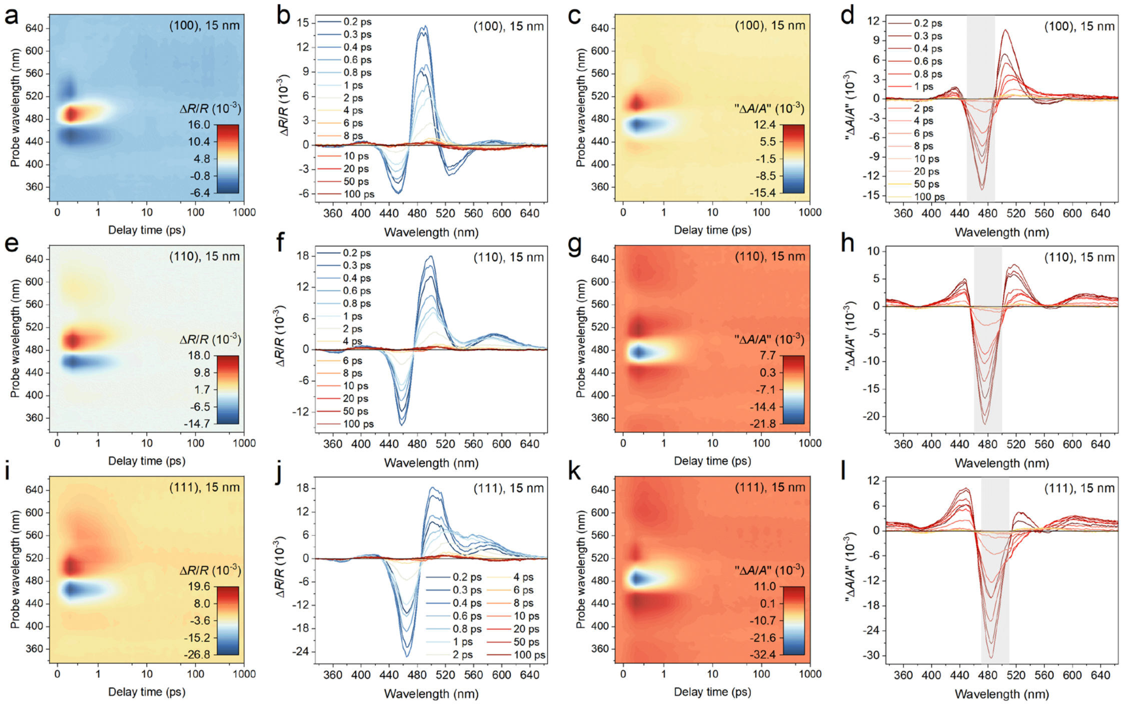

Supplementary Fig. 19 │ Transient reflection spectroscopy of 15 nm Cu_2O films. a-l, TR maps, TR spectra (left two columns) and the corresponding inverse Hilbert transformed TR maps and spectra (right two columns) of 15 nm Cu_2O films with crystal orientations of (100) (**a-d**), (110) (**e-h**), and (111) (**i-l**). Extraction spectral ranges are indicated with a grey background.

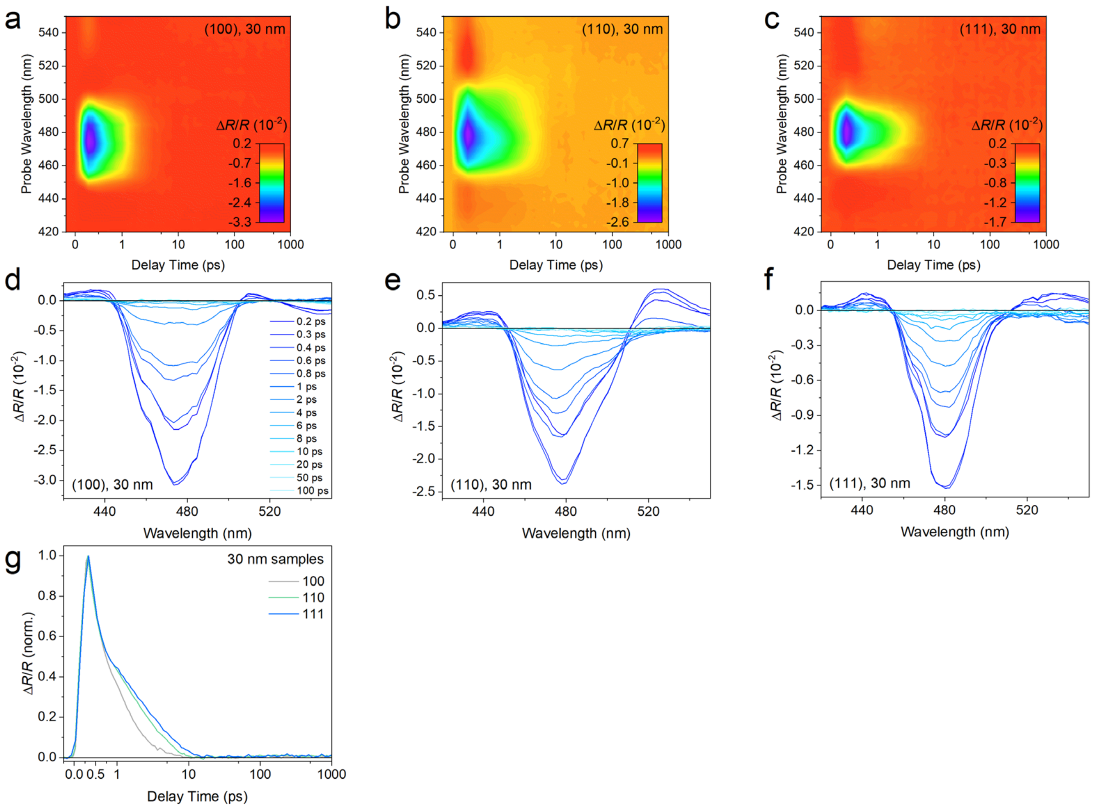

Supplementary Fig. 20 │ Transient reflection spectroscopy of 30 nm Cu_2O films. a-f, TR maps and corresponding spectra of 30 nm Cu_2O films with crystal orientations of (100) (**a, d**), (110) (**b, e**), and (111) (**c, f**). **g,** The normalized kinetics of the transient reflection signal of the (100) sample (450 – 500 nm), the (110) sample (460 – 500 nm) and the (111) sample (460 – 500 nm). All samples were photoexcited using a 3.1-eV pump with a fluence of ~110 μJ·cm^{-2}. For all samples, the first decay represents the carrier cooling process due to carrier-phonon interactions on a sub-picosecond timescale (0.31 ps for (100), 0.25 ps for (110) and (111) samples). The subsequent (second stage) decay has a lifetime of 1.28 ps, 2.47 ps and 3.25 ps for (100), (110) and (111) samples, respectively.

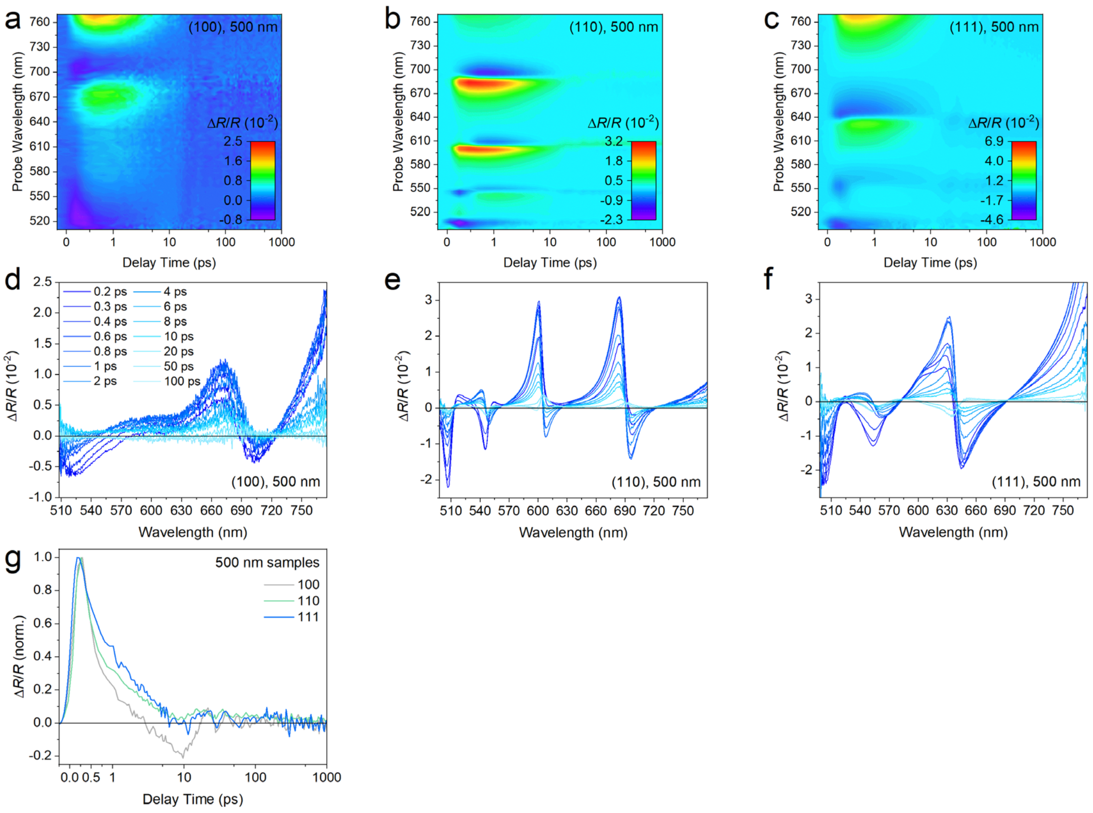

Supplementary Fig. 21 │ Transient reflection spectroscopy of 500 nm Cu_2O films. a-f, TR maps and corresponding spectra of 500 nm Cu_2O films with crystal orientations of (100) (**a, d**), (110) (**b, e**), and (111) (**c, f**). **g,** The normalized kinetics of the transient reflection signal of the (100) sample (510 – 550 nm), the (110) sample (500 – 512 nm) and the (111) sample (500 – 512 nm). All samples were photoexcited using a 3.1-eV pump with a fluence of ~255 $\mu J \cdot cm^{-2}$, corresponding to a carrier density of ~1.0×10^{19} cm^{-3}. The TR signals of 500-nm Cu_2O films have oscillations extending into the near-infrared region. We attribute these to the interaction of the light reflected from the front and back surfaces of the 500-nm films. Similar interference fringes were also observed in transition metal oxides[27], metal selenides[28], and halide perovskites[29]. Based on biexponential fitting for the recovery of the GSB signals in Supplementary Fig. 11g, the initial delay lifetime is 0.26 ps, 0.27 ps and 0.46 ps, and the second-stage decay lifetime is 1.39 ps, 2.43 ps and 3.41 ps for the (100), (110) and (111) samples, respectively.

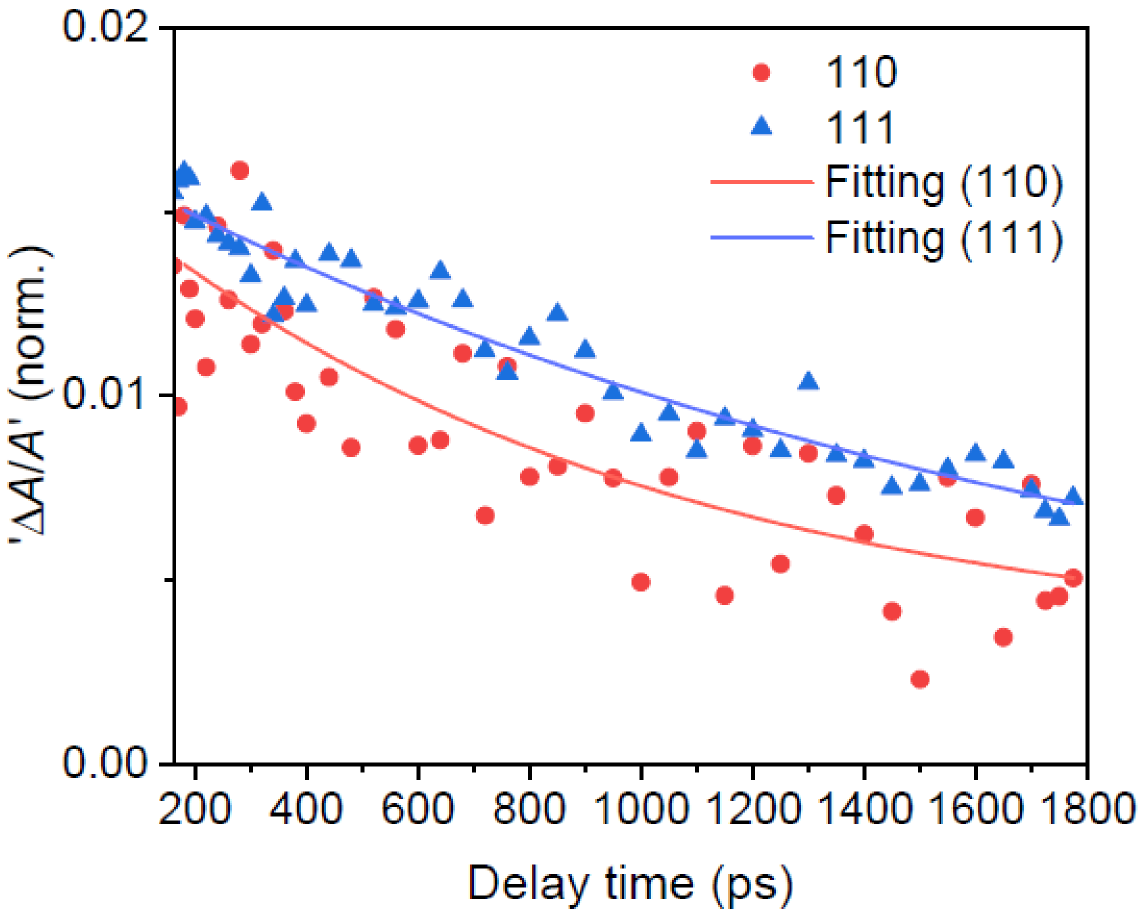

Supplementary Fig. 22 | Third-stage GSB decay of the 15-nm (110) and (111) samples extracted from the Hilbert transform of transient reflection signals.

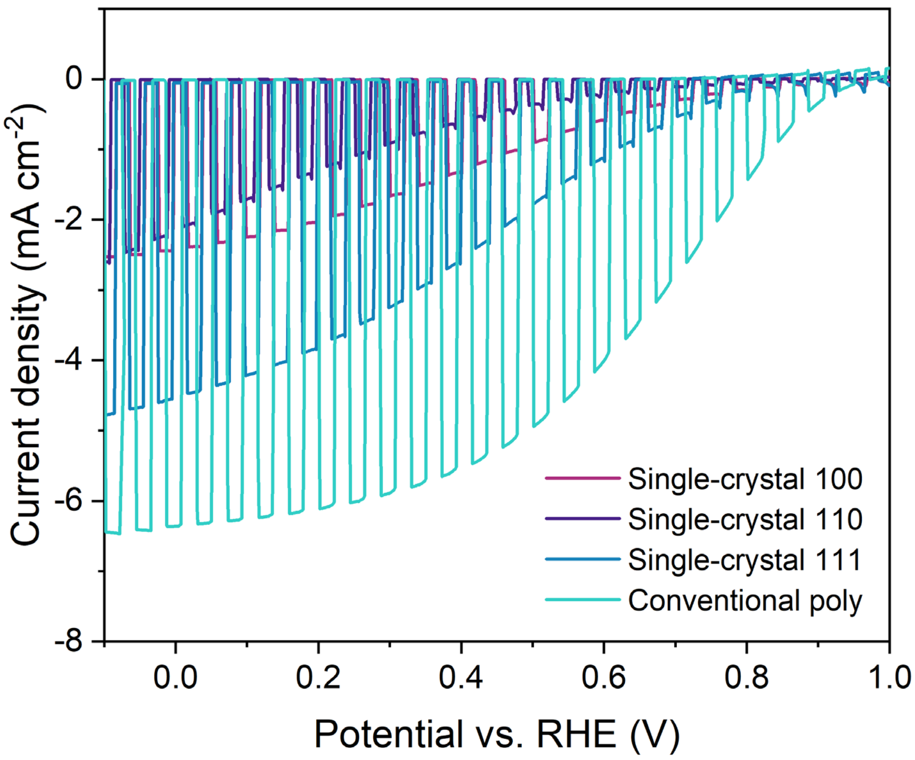

Supplementary Fig. 23 │ Current density-potential (*j-E*) responses of SC-Cu_2O and the conventional polycrystalline photocathodes**.** The photocathodes were tested in a pH 5 buffered electrolyte under the simulated one-sun illumination.

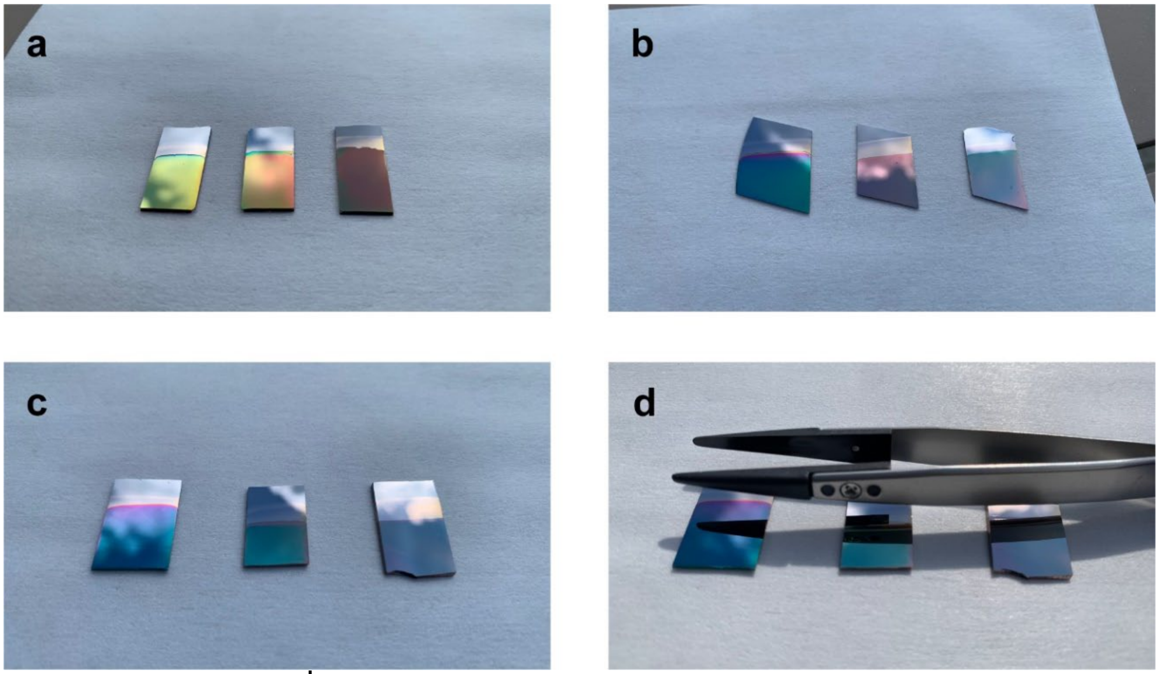

Supplementary Fig. 24 │ Photos of SC-Cu_2O thin films. a, The photo of SC-Cu_2O (100) with various thicknesses. **b**, The photo of SC-Cu_2O (110) with various thicknesses. **c**, The photo of SC-Cu_2O (111) with various thicknesses. **d**, The photo of a pair of tweezers on single-crystal thin films with a sharp reflection.

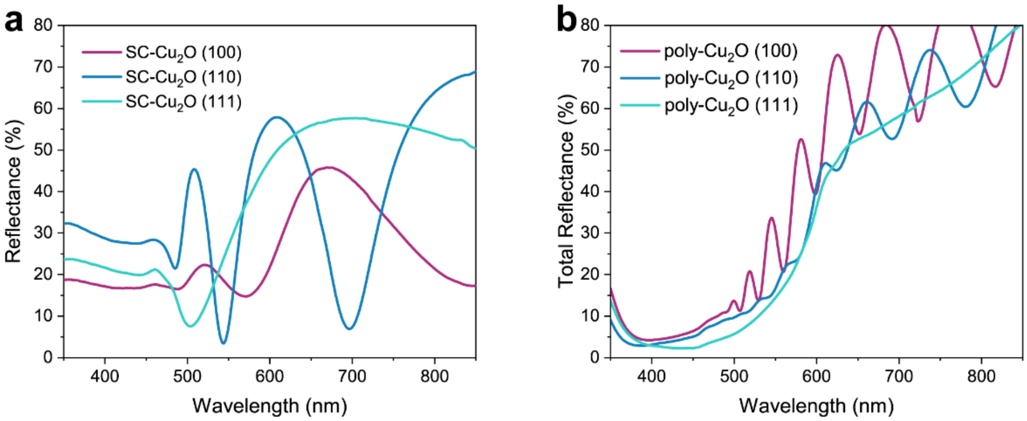

Supplementary Fig. 25 | UV-vis total reflectance spectra on the single-crystal Cu_2O (50 min) and poly-Cu_2O thin films with various crystal orientations. The measurement was carried out using an integration sphere with a double-beam configuration.

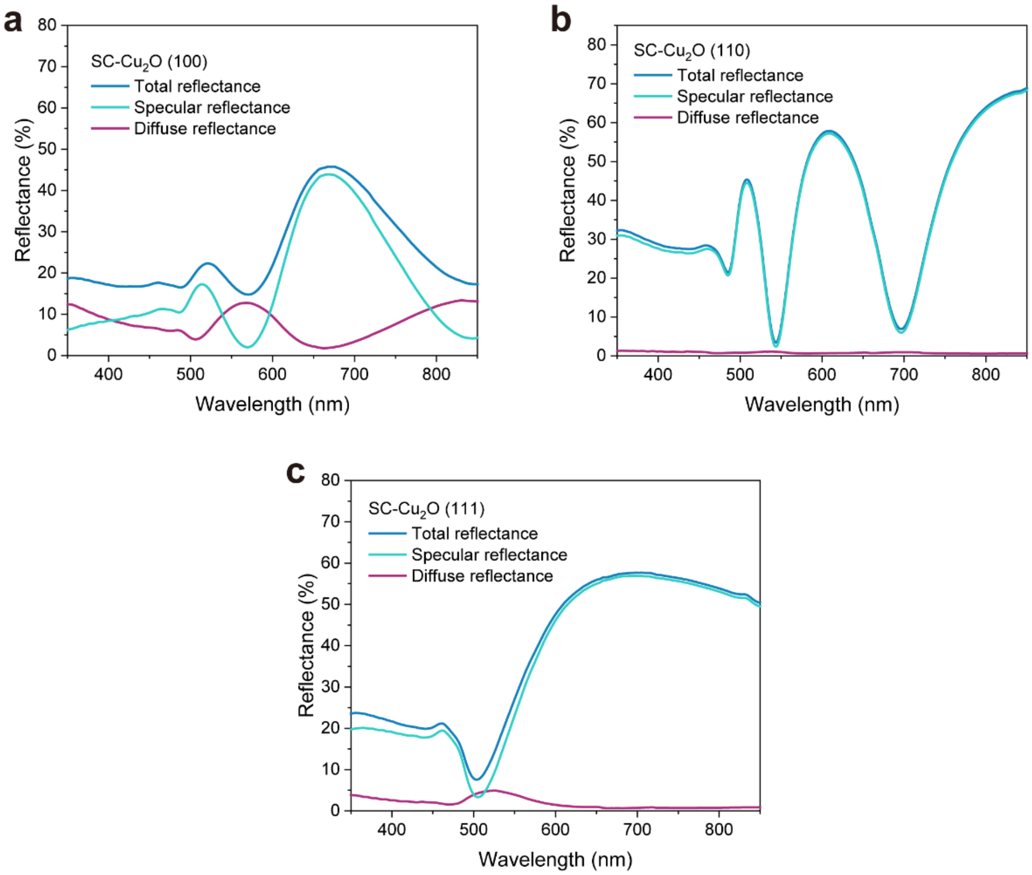

Supplementary Fig. 26 | UV-vis reflectance spectra. a-c, Reflectance spectra on the single-crystal Cu_2O thin films of (100) (**a**), (110) (**b**) and (111) (**c**) using an integration sphere with the double-beam configuration. Diffuse and total reflectance were distinguished by shifting the detecting angles between the detector and the sample surface.

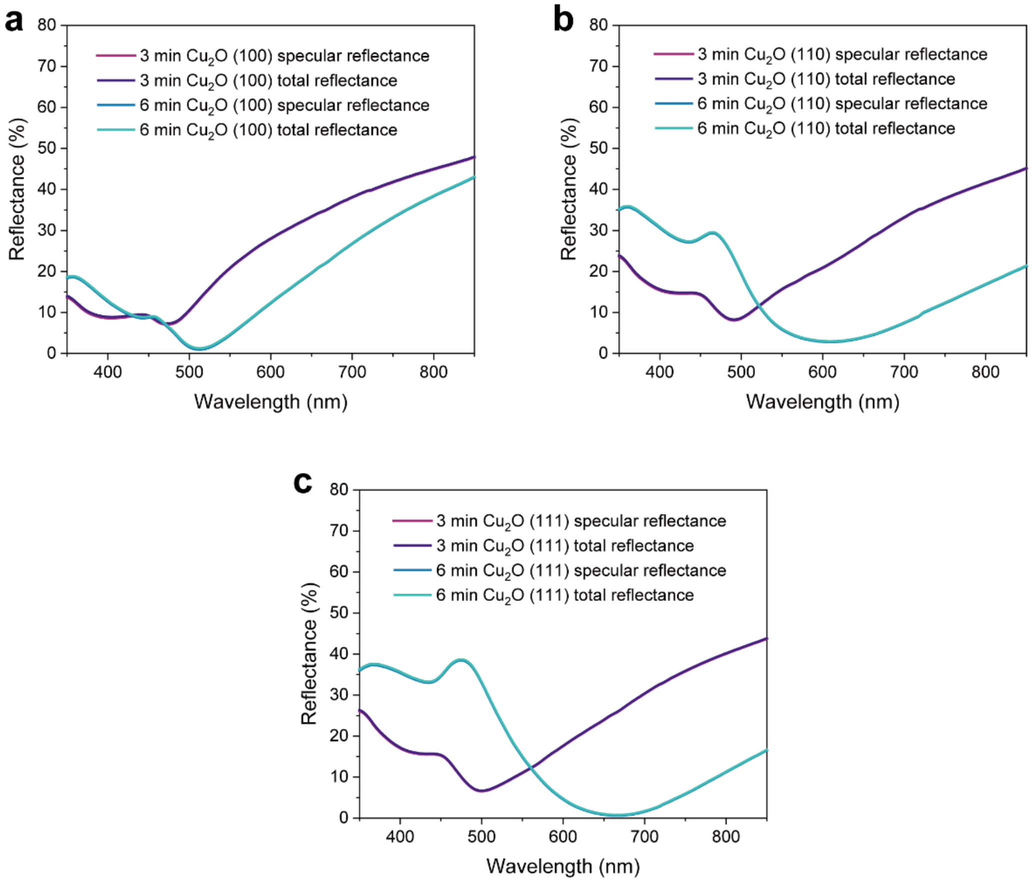

Supplementary Fig. 27 | UV-vis reflectance spectra. a-c, Reflectance spectra on the single-crystal Cu_2O thin films of (100) (**a**), (110) (**b**) and (111) (**c**) using an integration sphere with the double-beam configuration. Two thicknesses (~13.5 nm and ~27 nm) were tested for each crystal orientation.

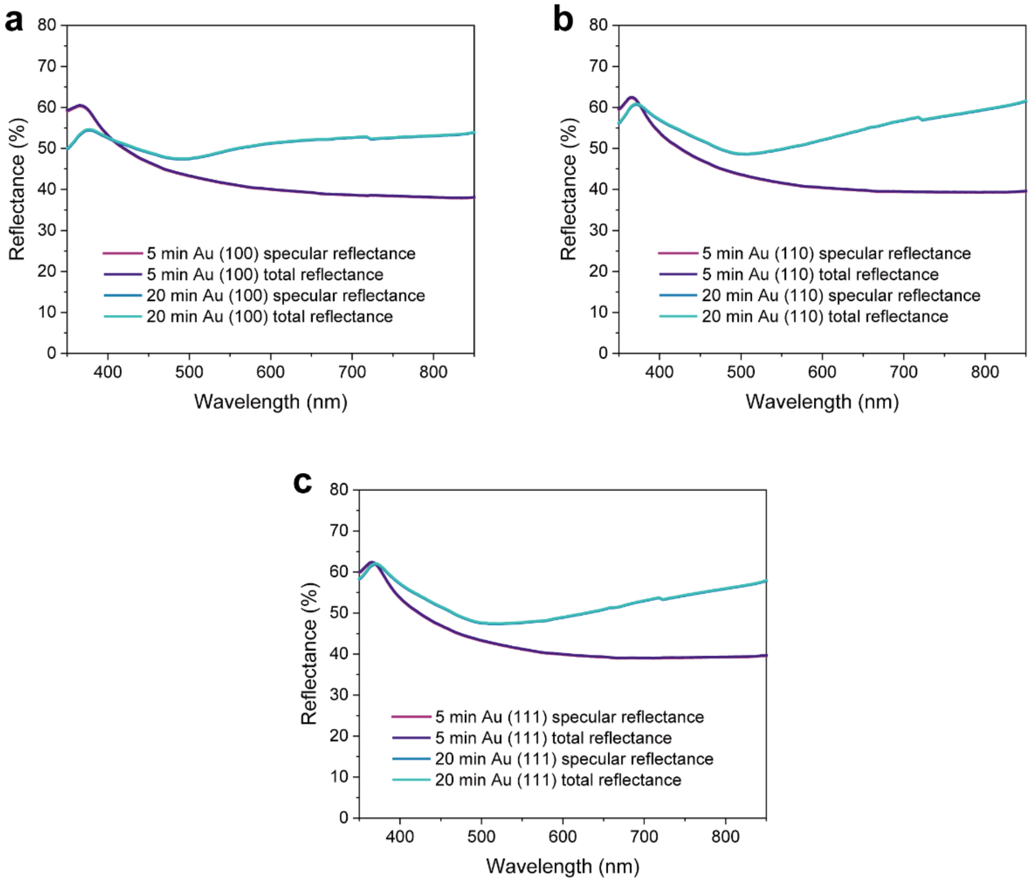

Supplementary Fig. 28 │ UV-vis reflectance spectra. a-c, Reflectance spectra on the Au buffer layers of (100) (**a**), (110) (**b**) and (111) (**c**) using an integration sphere with the double-beam configuration.

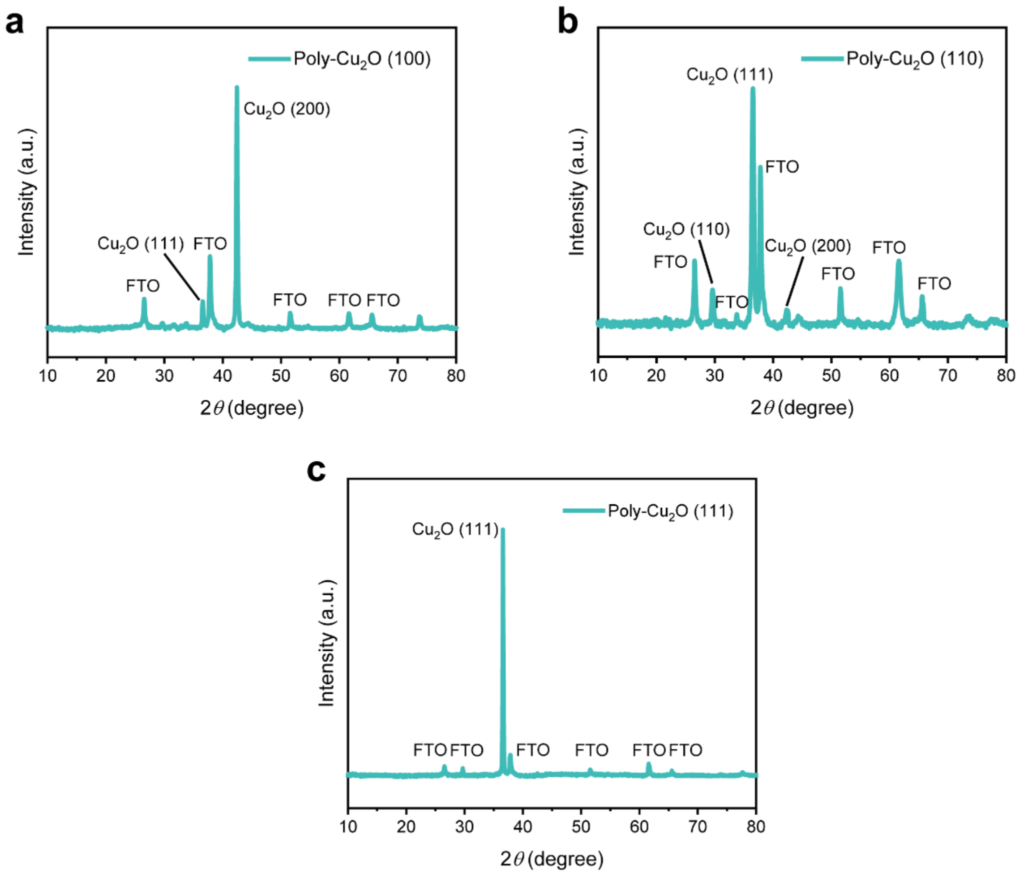

Supplementary Fig. 29 │ X-ray diffraction patterns. a-c, XRD patterns on the Poly-Cu_2O thin films on FTO glass substrates of dominant (100) (**a**), (110) (**b**) and (111) (**c**) orientations.

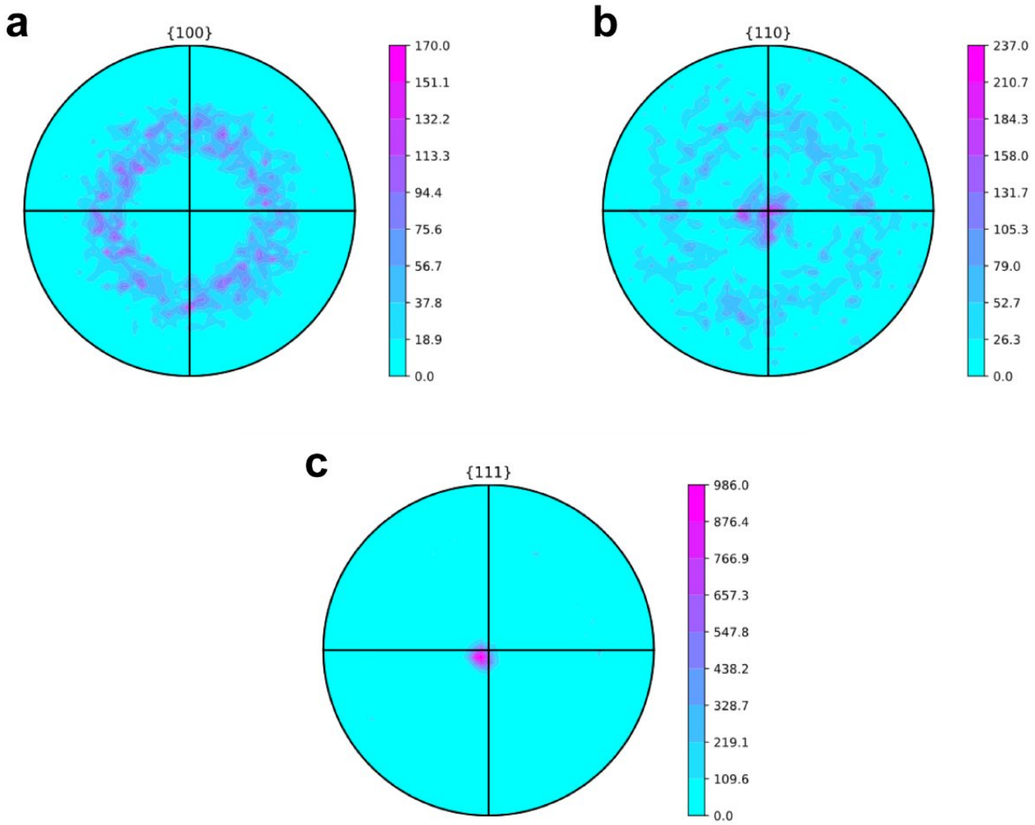

Supplementary Fig. 30 │ Pole figures on polycrystalline Cu_2O films. a-c, Pole figures for polycrystalline Cu_2O films with dominant (100) (**a**), (110) (**b**) and (111) (**c**) orientations prepared by modified electrochemical deposition.

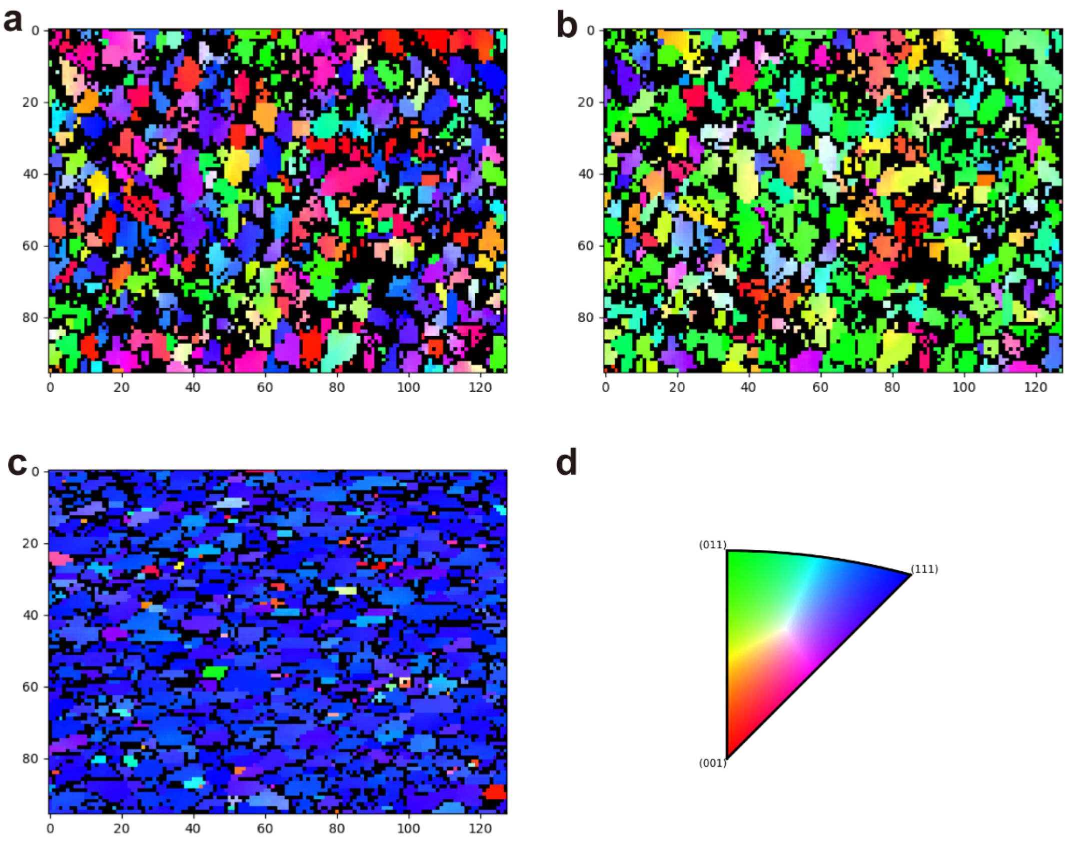

Supplementary Fig. 31 │ EBSD z-IPF maps. a-c, z-IPF maps showing dominant crystal orientations in the polycrystalline Cu_2O films with dominant (100) (**a**), (110) (**b**) and (111) (**c**) orientations. **d**, Colour indicator for crystal orientations.

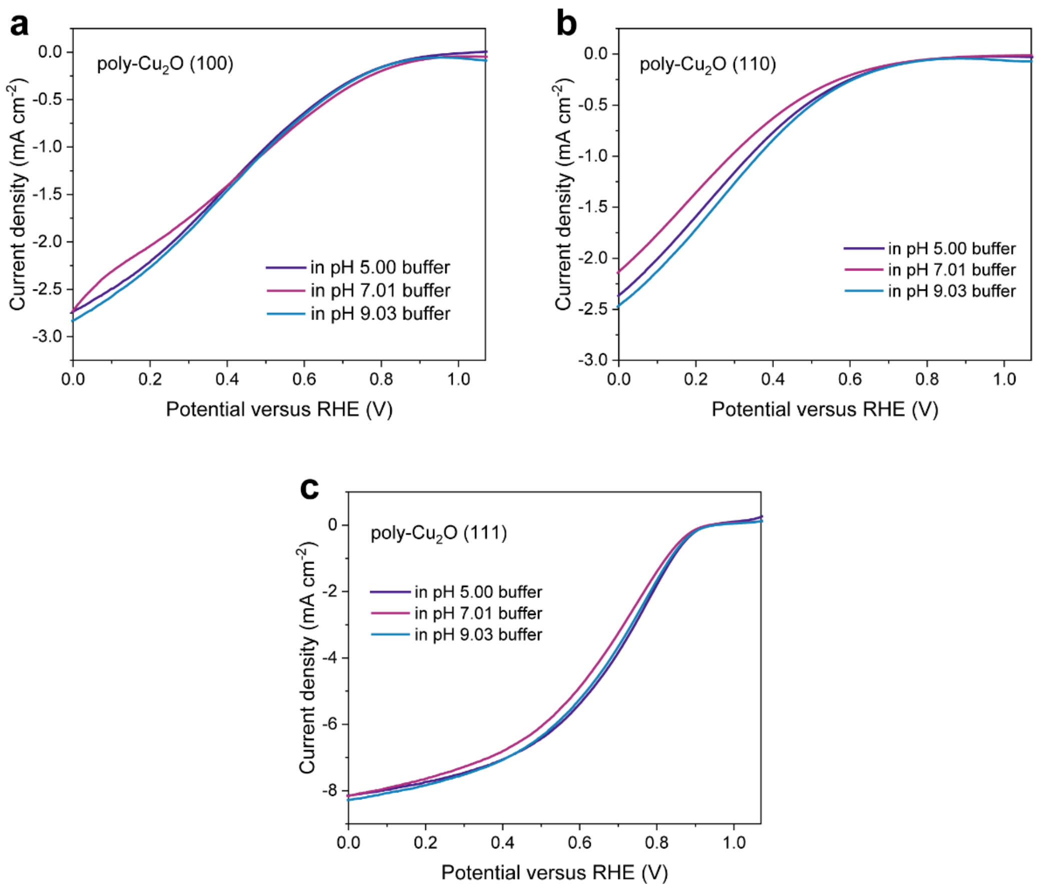

Supplementary Fig. 32 │ The pH-dependent photoelectrochemical performance. a-c, *j-V* curves of poly-Cu_2O of (100) (**a**), (110) (**b**) and (111) (**c**) for photoelectrochemical water reduction in acidic, neutral and alkaline electrolytes.

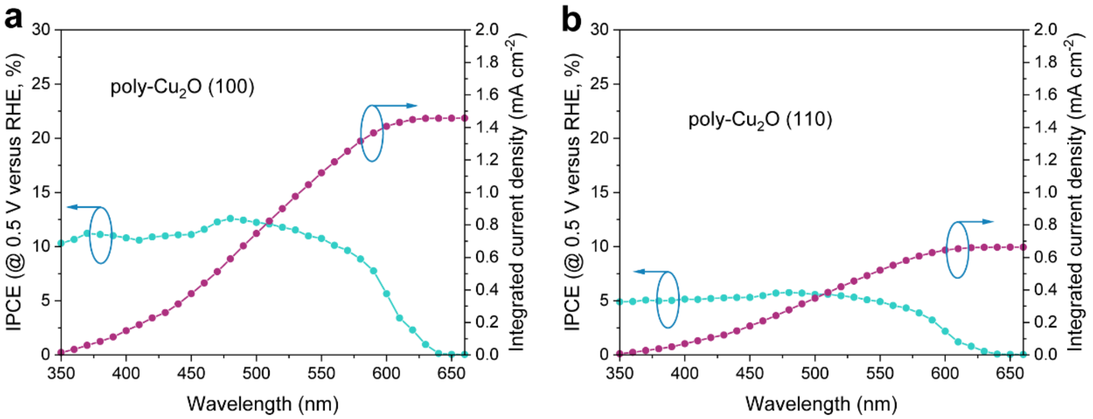

Supplementary Fig. 33 │ Wavelength-dependent IPCE of poly-Cu_2O photocathodes. a, b, Wavelength-dependent IPCE and integrated current density of poly-Cu_2O of (100) (**a**) and (110) (**b**) crystal orientations. All photocathodes are tested in pH 5 electrolyte with applied bias of 0.5 V versus RHE.

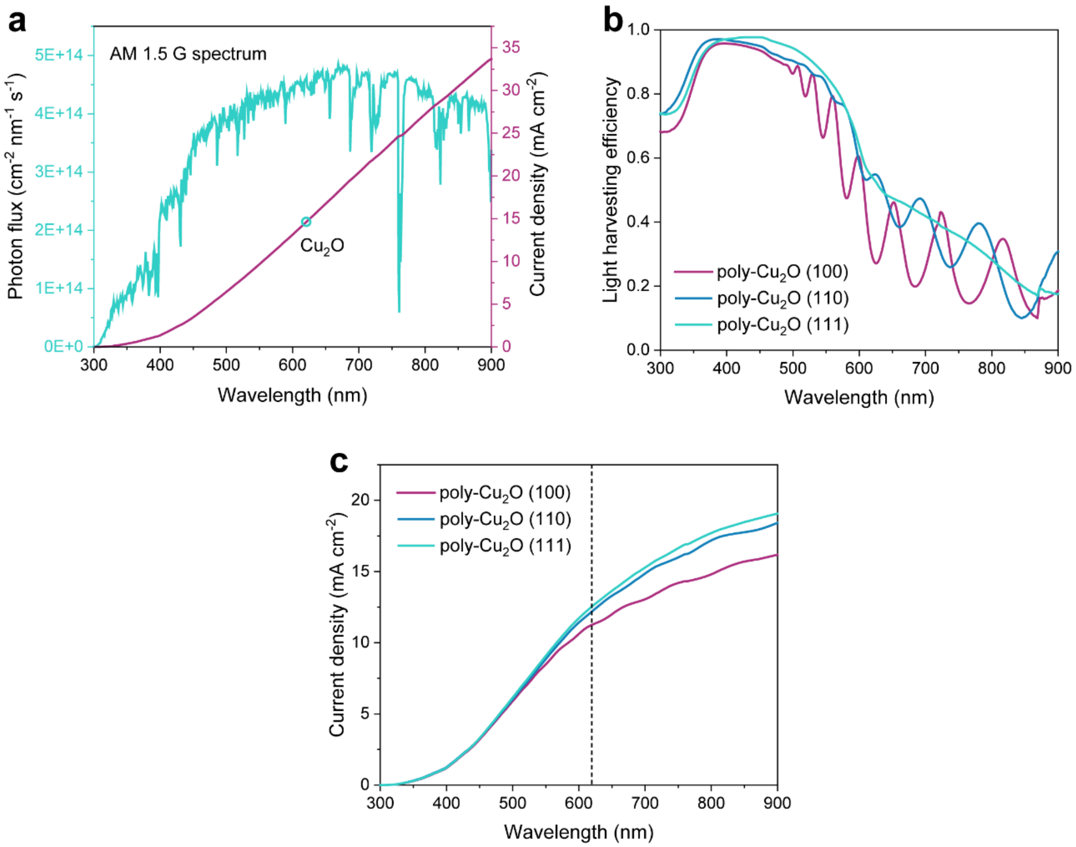

Supplementary Fig. 34 | The current flux calculation for polycrystalline Cu_2O photocathodes. a, The spectrum of AM1.5G with wavelength-dependent integrated current density assuming 100% incident photon-to-current conversion efficiency. **b**, The absorptance spectra of various poly-Cu_2O photocathodes. **c**, Wavelength-dependent integrated current density assuming 100% absorbed photon-to-current conversion efficiency.

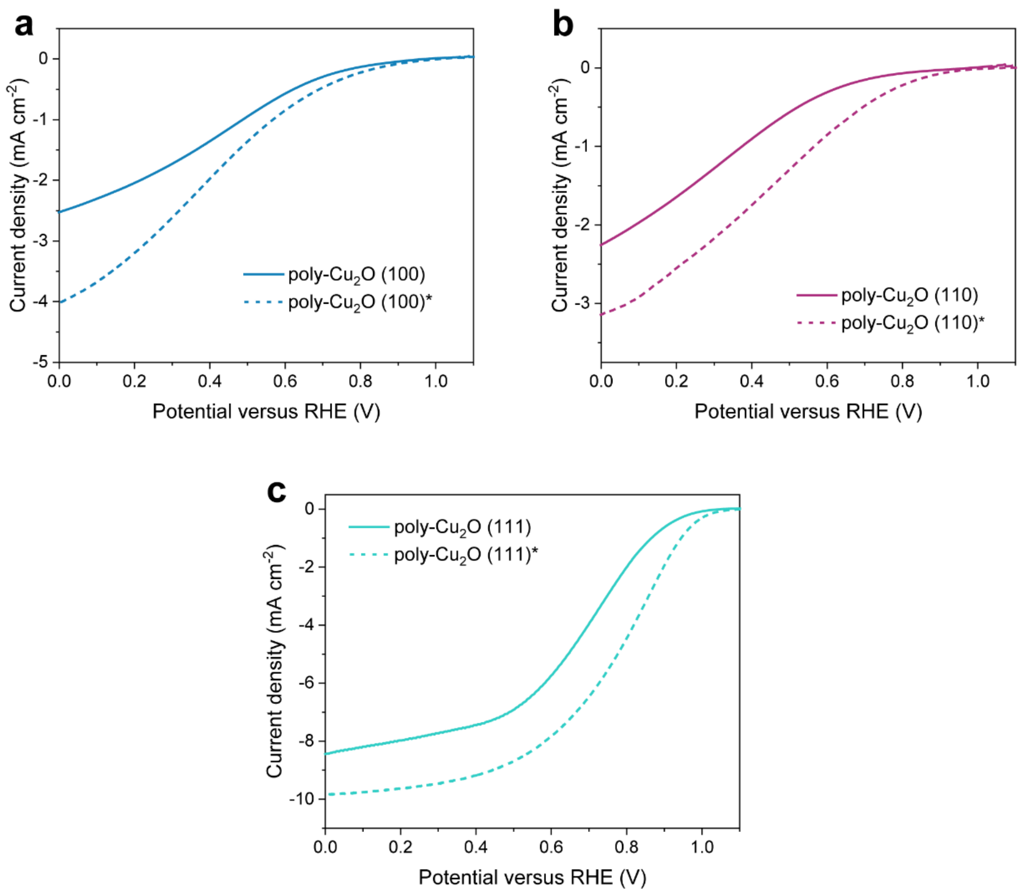

Supplementary Fig. 35 │ Photoelectrochemical performance with and without a sacrificial agent. a-c, *j*-*V* curves of poly-Cu_2O of (100) (**a**), (110) (**b**) and (111) (**c**) for photoelectrochemical water (solid) and sacrificial agent (dashed) reduction.

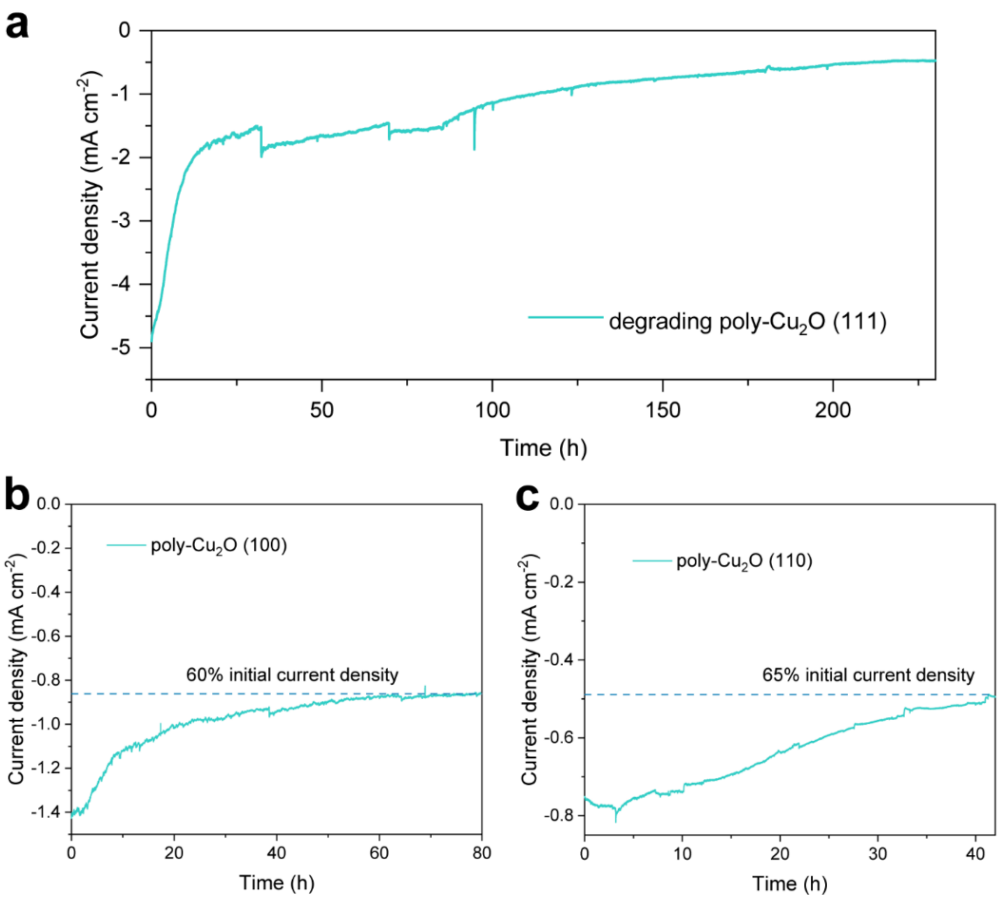

Supplementary Fig. 36 │ Stability tests on poly-Cu_2O photocathodes. a, Extended stability test on the poly-Cu_2O (111) photocathode with 100 nm TiO_2 protection layer at the fixed bias of 0.5 V versus RHE in pH 5 buffered electrolyte under simulated one-sun illumination (air mass 1.5 G spectrum) after initial current density drop of 15%. **b, c**, Stability test on poly-Cu_2O (100) (**b**) and poly-Cu_2O (110) (**c**) with the same condition of testing poly-Cu_2O (111) photocathodes.

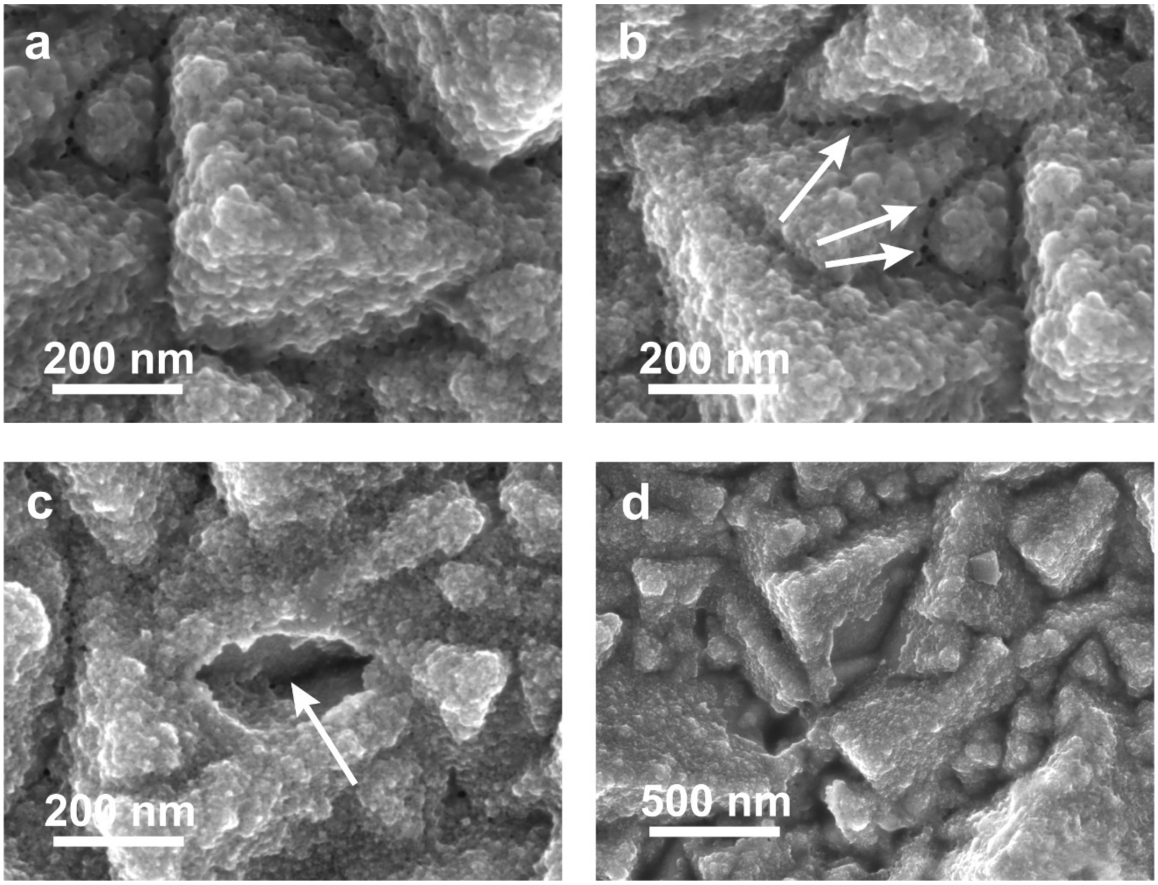

Supplementary Fig. 37 │ Top-view SEM images. a-d, SEM images of polycrystalline Cu_2O photocathodes after photoelectrochemical stability tests with arrows showing “pinhole” growth on the corroded TiO_2 layers.

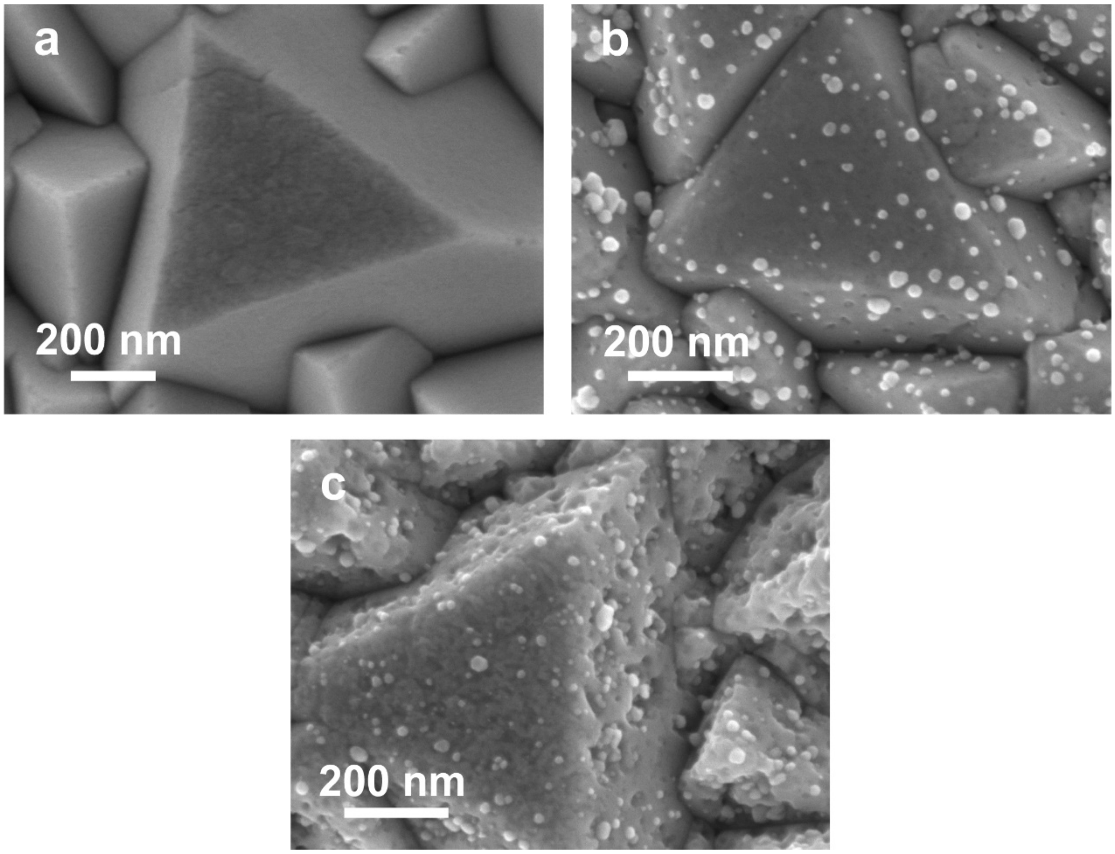

Supplementary Fig. 38 | Top-view scanning electron microscopy (SEM) images. a-c, SEM images of bare polycrystalline Cu_2O thin film after chronoamperometry test in pH 7 buffer under simulated AM 1.5G illumination for 0 (**a**), 20 (**b**) and 120 s (**c**). White spots are Cu particles.

References

1. Malerba, C. *et al.* Absorption coefficient of bulk and thin film Cu_2O. *Solar Energy Materials and Solar Cells* **95**, 2848–2854 (2011).
2. Pan, L. *et al.* Boosting the performance of Cu_2O photocathodes for unassisted solar water splitting devices. *Nat Catal* **1**, 412–420 (2018).
3. Pan, L. *et al.* Cu_2O photocathodes with band-tail states assisted hole transport for standalone solar water splitting. *Nat Commun* **11**, 318 (2020).
4. Paracchino, A., Laporte, V., Sivula, K., Grätzel, M. & Thimsen, E. Highly active oxide photocathode for photoelectrochemical water reduction. *Nature Mater* **10**, 456–461 (2011).
5. Dias, P. *et al.* Transparent Cuprous Oxide Photocathode Enabling a Stacked Tandem Cell for Unbiased Water Splitting. *Adv. Energy Mater.* **5**, 1501537 (2015).
6. Luo, J. *et al.* Cu_2O Nanowire Photocathodes for Efficient and Durable Solar Water Splitting. *Nano Lett.* **16**, 1848–1857 (2016).
7. Minami, T., Nishi, Y. & Miyata, T. Efficiency enhancement using a $Zn_{1-x}Ge_x$-O thin film as an n-type window layer in Cu_2O-based heterojunction solar cells. *Appl. Phys. Express* **9**, 052301 (2016).
8. Niu, W. *et al.* Extended Light Harvesting with Dual Cu_2O-Based Photocathodes for High Efficiency Water Splitting. *Advanced Energy Materials* **8**, 1702323 (2018).
9. Minami, T., Nishi, Y., Miyata, T. & Nomoto, J. High-Efficiency Oxide Solar Cells with ZnO/Cu_2O Heterojunction Fabricated on Thermally Oxidized Cu_2O Sheets. *Appl. Phys. Express* **4**, 062301 (2011).
10. Horowitz, K. A., Remo, T. W., Smith, B. & Ptak, A. J. *A Techno-Economic Analysis and Cost Reduction Roadmap for III-V Solar Cells.* NREL/TP--6A20-72103, 1484349 http://www.osti.gov/servlets/purl/1484349/ (2018) doi:10.2172/1484349.
11. Čulík, P. *et al.* Design and Cost Analysis of 100 MW Perovskite Solar Panel Manufacturing Process in Different Locations. *ACS Energy Lett.* **7**, 3039–3044 (2022).

12. Petrus, M. L., Bein, T., Dingemans, T. J. & Docampo, P. A low cost azomethine-based hole transporting material for perovskite photovoltaics. *J. Mater. Chem. A* **3**, 12159–12162 (2015).

13. Zheng, M. *et al.* III-Vs at scale: a PV manufacturing cost analysis of the thin film vapor-liquid-solid growth mode: III-Vs at scale: a PV manufacturing cost analysis. *Prog. Photovolt: Res. Appl.* **24**, 871–878 (2016).

14. Kim, J. H., Hansora, D., Sharma, P., Jang, J.-W. & Lee, J. S. Toward practical solar hydrogen production – an artificial photosynthetic leaf-to-farm challenge. *Chem. Soc. Rev.* **48**, 1908–1971 (2019).

15. Dharmadasa, I. M. & Haigh, J. Strengths and Advantages of Electrodeposition as a Semiconductor Growth Technique for Applications in Macroelectronic Devices. *J. Electrochem. Soc.* **153**, G47 (2005).

16. Chen, H., Wei, Z., Zheng, X. & Yang, S. A scalable electrodeposition route to the low-cost, versatile and controllable fabrication of perovskite solar cells. *Nano Energy* **15**, 216–226 (2015).

17. Zou, X. *et al.* Electrodeposition of crystalline silicon films from silicon dioxide for low-cost photovoltaic applications. *Nat Commun* **10**, 5772 (2019).

18. Brandt, I. S., Tumelero, M. A., Pelegrini, S., Zangari, G. & Pasa, A. A. Electrodeposition of Cu_2O: growth, properties, and applications. *J Solid State Electrochem* **21**, 1999–2020 (2017).

19. Pinarbasi, M. *et al.* ROLL TO ROLL MANUFACTURING OF FLEXIBLE CIGS CELLS AND PANELS. (2010).

20. Manivannan, R. & Victoria, S. N. Preparation of chalcogenide thin films using electrodeposition method for solar cell applications – A review. *Solar Energy* **173**, 1144–1157 (2018).

21. Mizuno, K. *et al.* Structural and Electrical Characterizations of Electrodeposited p-Type Semiconductor Cu_2O Films. *J. Electrochem. Soc.* **152**, C179 (2005).

22. Abdi, F. F. *et al.* Efficient solar water splitting by enhanced charge separation in a bismuth vanadate-silicon tandem photoelectrode. *Nat Commun* **4**, 2195 (2013).

23. Dotan, H., Sivula, K., Grätzel, M., Rothschild, A. & Warren, S. C. Probing the photoelectrochemical properties of hematite (α-Fe_2O_3) electrodes using hydrogen peroxide as a hole scavenger. *Energy Environ. Sci.* **4**, 958–964 (2011).

24. Grätzel, M. Photoelectrochemical cells. *Nature* **414**, 338–344 (2001).

25. Shi, X., Cai, L., Ma, M., Zheng, X. & Park, J. H. General Characterization Methods for Photoelectrochemical Cells for Solar Water Splitting. *ChemSusChem* **8**, 3192–3203 (2015).

26. Alwan, N. T., Shcheklein, S. E. & Ali, O. M. Evaluation of distilled water quality and production costs from a modified solar still integrated with an outdoor solar water heater. *Case Studies in Thermal Engineering* **27**, 101216 (2021).

27. Joly, A. G. *et al.* Carrier dynamics in α-Fe_2O_3 (0001) thin films and single crystals probed by femtosecond transient absorption and reflectivity. *Journal of Applied Physics* **99**, 053521 (2006).

28. Eid, J. *et al.* Ultrafast pump-probe reflectance spectroscopy: Why sodium makes $Cu(In,Ga)Se_2$ solar cells better. *Solar Energy Materials and Solar Cells* **140**, 33–37 (2015).

29. Yang, Y. *et al.* Top and bottom surfaces limit carrier lifetime in lead iodide perovskite films. *Nat Energy* **2**, 1–7 (2017).
